# Reversible Treatment of Pressure Overload‐Induced Left Ventricular Hypertrophy through *Drd5* Nucleic Acid Delivery Mediated by Functional Polyaminoglycoside

**DOI:** 10.1002/advs.202003706

**Published:** 2021-01-06

**Authors:** Xiaoliang Jiang, Meiyu Shao, Xue Liu, Xing Liu, Xu Zhang, Yuming Wang, Kunlun Yin, Shuiyun Wang, Yang Hu, Pedro A Jose, Zhou Zhou, Fu‐Jian Xu, Zhiwei Yang

**Affiliations:** ^1^ NHC Key Laboratory of Human Disease Comparative Medicine (The Institute of Laboratory Animal Sciences, CAMS & PUMC), and Beijing Engineering Research Center for Experimental Animal Models of Human Critical Diseases 5 Pan Jia Yuan Nan Li, Chaoyang District Beijing 100021 P. R. China; ^2^ Key Lab of Biomedical Materials of Natural Macromolecules Ministry of Education Beijing Laboratory of Biomedical Materials Beijing Advanced Innovation Center for Soft Matter Science and Engineering Beijing University of Chemical Technology Beijing 100029 P. R. China; ^3^ Department of Hepato‐Biliary‐Pancreatic Surgery Henan Provincial People's Hospital People's Hospital of Zhengzhou University Zhengzhou Henan 450003 P. R. China; ^4^ State Key Laboratory of Cardiovascular Disease Beijing Key Laboratory for Molecular Diagnostics of Cardiovascular Diseases Diagnostic Laboratory Service Fuwai Hospital National Center for Cardiovascular Diseases Chinese Academy of Medical Sciences and Peking Union Medical College Beijing 100037 P. R. China; ^5^ Department of Cardiovascular Surgery State Key Laboratory of Cardiovascular Disease Fuwai Hospital National Center for Cardiovascular Diseases Chinese Academy of Medical Sciences and Peking Union Medical College Beijing 100037 P. R. China; ^6^ Department of Pharmacology and Physiology The George Washington University School of Medicine & Health Sciences Washington DC 20052 USA; ^7^ Department of Medicine Division of Kidney Diseases & Hypertension The George Washington University School of Medicine & Health Sciences Washington DC 20052 USA

**Keywords:** autophagy, dopamine D5 receptor, hyperbranched polyaminoglycoside, left ventricular hypertrophy, reactive oxygen species

## Abstract

Left ventricular hypertrophy and fibrosis are major risk factors for heart failure, which require timely and effective treatment. Genetic therapy has been shown to ameliorate hypertrophic cardiac damage. In this study, it is found that in mice, the dopamine D5 receptor (D5R) expression in the left ventricle (LV) progressively decreases with worsening of transverse aortic constriction‐induced left ventricular hypertrophy. Then, a reversible treatment of left ventricular hypertrophy with *Drd5* nucleic acids delivered by tobramycin‐based hyperbranched polyaminoglycoside (SS‐HPT) is studied. The heart‐specific increase in D5R expression by SS‐HPT/*Drd5* plasmid in the early stage of left ventricular hypertrophy attenuates cardiac hypertrophy and fibrosis by preventing oxidative and endoplasmic reticulum (ER) stress and ameliorating autophagic dysregulation. By contrast, SS‐HPT/*Drd5* siRNA promotes the progression of left ventricular hypertrophy and accelerates the deterioration of myocardial function into heart failure. The reduction in cardiac D5R expression and dysregulated autophagy are observed in patients with hypertrophic cardiomyopathy and heart failure. The data show a cardiac‐specific beneficial effect of SS‐HPT/*Drd5* plasmid on myocardial remodeling and dysfunction, which may provide an effective therapy of patients with left ventricular hypertrophy and heart failure.

## Graphical abstract

Reversible treatments (deterioration or improvement) of pressure overload‐induced left ventricular hypertrophy and heart failure are readily achieved via one functional polyaminoglycoside vector (SS‐HPT) loaded with *Drd5* siRNA (SS‐HPT/*Drd5* siRNA) or *Drd5* plasmid (SS‐HPT/*Drd5* plasmid)

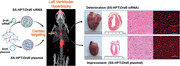

## 1 Introduction

Hypertension, with left ventricular hypertrophy and fibrosis, is a major risk factor for heart failure.^[^ ^1^ ^]^ Heart failure is associated with transcriptional changes in myocytes that contribute to contractile dysfunction, as well as an increase in fibrosis, ultimately leading to systolic or diastolic heart failure.^[^ ^2^, ^3^ ^]^ In the case of pressure overload, resulting from aortic stenosis or systemic arterial hypertension, the heart often develops an adaptive concentric hypertrophy.^[^ ^4^ ^]^ Concentric hypertrophy gradually progresses to eccentric hypertrophy and eventually to systolic heart failure, if the stress is sustained. During the transition from compensated hypertrophy to decompensated heart failure, severe fibrosis usually occurs concurrently with cardiomyocyte hypertrophy, cellular apoptosis, and inflammatory cell infiltration. However, the molecular mechanisms that mediate the development of left ventricular hypertrophy and the transition to heart failure are incompletely understood.^[^ ^5^ ^]^ A widely accepted strategy is positive intervention in the early stage of heart failure, by inhibiting the pathological left ventricular hypertrophy.^[^ ^6^ ^]^ A meta‐analysis of the long‐term outcome (about 7 years) of medical therapy‐resistant obstructive cardiac hypertrophy showed that the mortality with alcohol septal ablation and myectomy was low, 1.5% and 1.4%, respectively.^[^ ^7^ ^]^ However, in a study with a longer follow‐up of 20 years, the mortality after septal myectomy in patients with cardiac hypertrophy was 16%, 56% of which were cardiac‐related.^[^ ^8^ ^]^ Therefore, targeting key causative drivers of the development and progression of left ventricular hypertrophy is urgent, which may lead to improvement of clinical treatment.

Dopamine D5 receptor (D5R), one of the five subtypes of dopamine receptors, exhibits constitutive activity.^[^ ^9^ ^]^ The chromosomal locus of D5R and its pseudo genes are linked to human essential hypertension.^[^ ^10^, ^11^, ^12^, ^13^ ^]^ Global D5R knockout, in mice, leads to high blood pressure and compensatory left ventricular hypertrophy.^[^ ^14^, ^15^ ^]^ Cardiac‐specific expression of a mutant human D5R mutation, in mice, causes dilated cardiomyopathy and dysfunction, independent of blood pressure.^[^ ^16^ ^]^ These findings indicate that the D5R is crucial in maintaining cardiac function; delivery of cardiac‐specific D5R may be a novel and effective gene therapeutic strategy for improving cardiac function. Gene therapy has been shown to ameliorate hypertrophic cardiac damage. Nucleic acid cannot be delivered directly into the heart, owing to its own instability and confounded by the complex environment in vivo.^[^ ^17^, ^18^, ^19^, ^20^ ^]^ However, because of the continuous flow of blood into the heart chambers, specifically formulated nucleic acid delivery vehicles are essential for effective gene therapy. Gene delivery vectors mainly involve viral and non‐viral agents.^[^ ^21^ ^]^ Viral vectors, such as lentivirus or adeno‐associated viral vectors, which are used for gene delivery, may suffer from limited packaging capacity, immunogenicity, and carcinogenesis risk.^[^ ^21^, ^22^ ^]^ By contrast, low host immunogenic non‐viral vectors, especially, polycation‐based vectors, have advantages over viral vectors because of properties, such as flexibility, ease of preparation, and large gene payload.^[^ ^23^, ^24^, ^25^, ^26^ ^]^ In particular, non‐viral‐mediated delivery systems have made advances in disease therapy due to their potency, vascular permeability, and high concentrations in the circulation.^[^ ^27^ ^]^

**Scheme 1:**
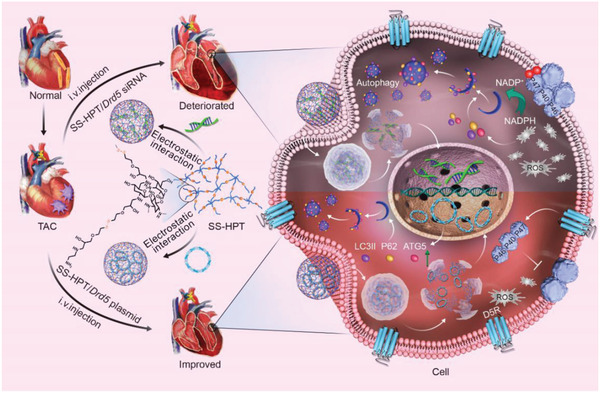
Illustration of the mechanism of the deterioration and improvement of left ventricular hypertrophy, mediated by functional polyaminoglycoside vectors (SS‐HPT) loaded with *Drd5* siRNA (SS‐HPT/*Drd5* siRNA) or *Drd5* plasmid (SS‐HPT/*Drd5* plasmid), respectively.

The tobramycin‐based hyperbranched polyaminoglycoside (SS‐HPT), composed of hydroxyl groups and disulfide bonds, has great biocompatibility and exhibits high gene delivery in different cell lines,^[^ ^28^, ^29^ ^]^ which could be an excellent delivery vector in vivo. In the present study, we found that cardiac D5R expression continuously decreased during the progression of left ventricular hypertrophy. We supposed that SS‐HPT coated with *Drd5* plasmid may protect the heart from transverse aortic constriction (TAC)‐induced left ventricular hypertrophy. SS‐HPT/*Drd5* plasmid complexes were constructed to improve the outcome of left ventricular hypertrophy (**Scheme** **1**), whereas SS‐HPT/*Drd5* siRNA served to deteriorate the outcome. In addition, our collected heart samples showed a trend in a decrease in cardiac D5R expression in patients with heart failure and those with a presumptive diagnosis of hypertrophic cardiomyopathy (HCM). This study shows that SS‐HPT/*Drd5* plasmid complexes may be a therapeutic strategy for the clinical treatment of patients with heart failure.

## 2 Results and Discussion

### 2.1 Characterization of Functional Polyaminoglycoside (SS‐HPT)

**Figure 1:**
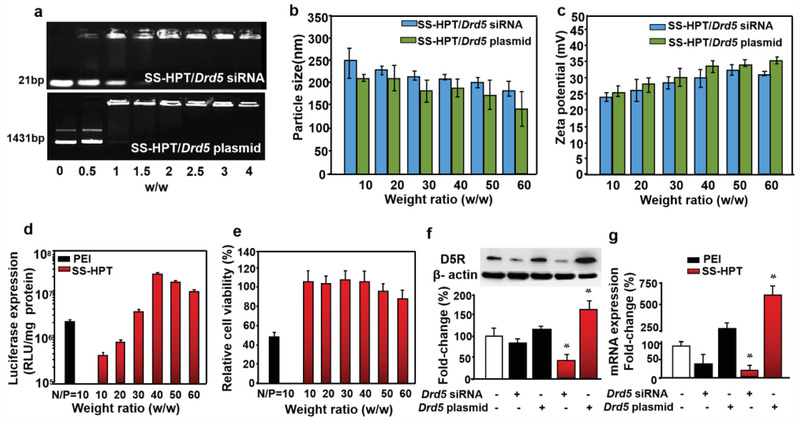
Biophysical properties and in vitro gene expression assay of SS‐HPT/*Drd5* complex. a) Electrophoretic mobility retardation assay of *Drd5* siRNA and *Drd5* plasmid in the complexes with SS‐HPT at various weight ratios. b) Particle size and c) *ζ*‐potential of SS‐HPT/*Drd5* siRNA and SS‐HPT/*Drd5* plasmid complexes at various weight ratios. d) Luciferase gene transfection efficiencies of SS‐HPT at various weight ratios in comparison with those mediated by polyethylenimine (PEI, 25 kDa), at its optimal N/P ratio of 10 in H9c2 cells. e) Relative cell viability (cytotoxicity) of PEI (black bar) and SS‐HPT (red bars) in H9c2 cells. (*n* = 6 per group). f) D5R protein expression (western blot) delivered by SS‐HPT (red bars) and PEI (black bars) in the H9c2 cells; and quantitative analysis used *β*‐actin for normalization (*n* = 8 per group, *p < 0.05 vs vehicle); white bar = vehicle only. g) D5R mRNA expression (RT‐qPCR) delivered by PEI and SS‐HPT in the H9c2 cells; quantitative analysis used *β*‐actin for normalization (*n* = 8 per group, *p < 0.05 vs control), one‐way ANOVA, Holm‐Sidak test. Data are expressed as mean ± SD.

SS‐HPT can compress nucleic acids (NA) to form nanocomplexes with appropriate particle sizes and surface charges via electrostatic interactions. We used agarose gel electrophoresis (**Figure** **1**a), dynamic light scattering (Figure 1b,c), and atomic force microscopy (AFM) imaging (Figure S1, Supporting Information) to verify the condensation of SS‐HPT/NA complexes. As shown in Figure 1a, SS‐HPT completely condensed NA at the weight ratio of two. The particle sizes and zeta potentials of SS‐HPT/NA at various weight ratios are shown in Figure 1b,c. All SS‐HPT/NA complexes with positive charge had particle sizes ranging from 100 to 300 nm, which can be internalized into the cell by means of clathrin‐dependent endocytosis (≈200 nm), as well as clathrin‐independent (≈90 nm) endocytosis, and micropinocytosis (>1 µm).^[^ ^25^, ^30^, ^31^ ^]^ Delivery of NA without SS‐HPT did not change D5R expression (Figure S2a, Supporting Information).With the increase in weight ratio, more polycations could compress NA, which made the condensation effect tighter and the complexes smaller, as well. During the incubation in a medium containing 10% FBS for 5 h (Figure S2b, Supporting Information), the sizes of SS‐HPT/NA complexes (at the weight ratio of 40) were almost unchanged, which demonstrated a good serum‐tolerant ability and stability of SS‐HPT/NA in the circulatory system.^[^ ^27^ ^]^ When the weight ratios increased, the *ζ*‐potentials of complexes also increased from 24 to 35 mV (Figure 1c), which provided an appropriate affinity between complexes and negatively charged cell membranes to improve the efficiency of cellular uptake. To confirm the ability to condense with a specific morphology, visualized images of SS‐HPT/NA complexes at the typical weight ratio of 40 were captured using AFM (Figure S1a,b, Supporting Information). The SS‐HPT/NA complexes exhibited relatively uniform nanospheres but became irregular after treatment with NaBH_4_ (Figure S1c,d, Supporting Information). Due to the reducibility of disulfide, SS‐HPT could be degraded into small molecules, which may have good biocompatibility and promote the resultant release of NA.

Low cytotoxicity and high transfection efficiency are two key factors that decide the success of a gene delivery system. To investigate the in vitro gene transfection efficiency of the SS‐HPT/pDNA complex, pRL‐CMV was used as the reporter gene in H9c2 cells and compared with polyethyleneimine (PEI) (with its optimal PEI nitrogen to plasmid DNA phosphate ratio (N/P) of 10), confirmed in Figure 1d and Figure S3, Supporting Information. SS‐HPT‐mediated transfection efficiency continuously increased until the weight ratio of 40; thereafter, the transfection efficiency slightly decreased with increasing weight ratio. In H9c2 cells, the transfection efficiency of SS‐HPT at its optimal weight ratio 40 was higher than that of PEI (25 kDa) at its optimal N/P ratio. Another essential index, cell viability, was determined by kit‐8 assay, and showed that SS‐HPT exhibited a concentration‐dependent minimal cytotoxicity in H9c2 cells (Figure 1e), which was similar to the findings in our previous work.^[^ ^28^, ^29^ ^]^ Compared with PEI (at the N/P ratio of 10), SS‐HPT showed lower cytotoxicity (at weight ratios ranging from 10 to 60) (Figure 1e).

**Figure 2:**
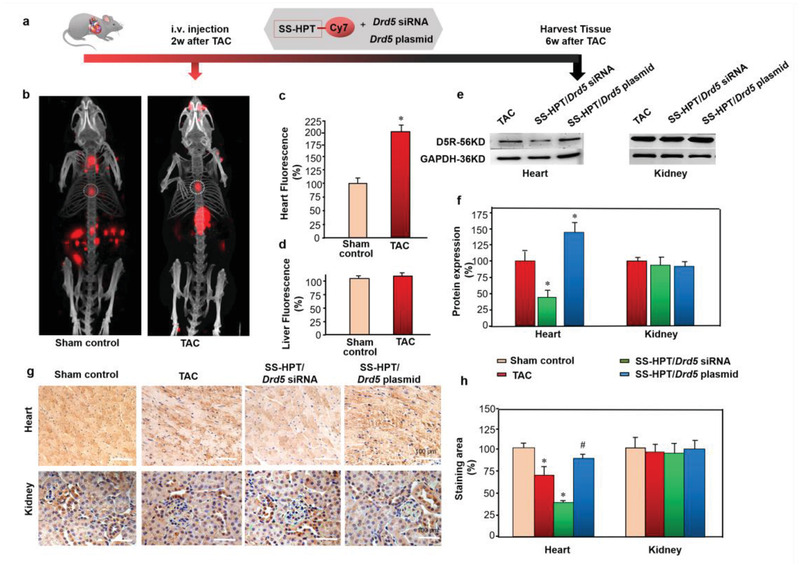
In vivo distribution of SS‐HPT/*Drd5* complex in TAC mice with left ventricular hypertrophy. a) Protocol for fluorescence emission tomography of cardiac expression by SS‐HPT/*Drd5* complex treatment in vivo. SS‐HPT was connected with CY7, then mixed with *Drd5* plasmid or *Drd5* siRNA, and injected in the second week in TAC and Sham mice; heart was harvested at 6 weeks after TAC surgery in C57BL/6J mice. b) SS‐HPT/*Drd5* complex location measured by fluorescence emission tomography (*n* = 3). c,d) Immunofluorescence of heart and liver in TAC and control mice (n = 3 per group, *p < 0.05 vs Sham control). e,f) D5R protein expression in the heart and kidney from TAC and control mice 4 weeks post SS‐HPT/*Drd5* complex injection; quantitative analysis used GAPDH for normalization (*n* = 8 per group, *p < 0.01 vs mock‐treated TAC mice). g,h) Representative images and quantitative analysis of *Drd5*‐positive‐stained heart and kidney from the control, TAC mice without and with SS‐HPT/*Drd5* siRNA, and SS‐HPT/*Drd5* plasmid treatment (*n* = 10/group, *p < 0.01 vs Sham control, #*p* < 0.05 vs SS‐HPT/*Drd5* siRNA and TAC); one‐way ANOVA, Holm‐Sidak test. Data are presented as the mean ±SD. TAC, transverse aortic constriction.

We used another reporter gene, plasmid EGFP‐N1 (pEGFP), to confirm further the efficacy of gene delivery with SS‐HPT in H9c2 cell. As shown in Figure S4, Supporting Information, the percentage of EGFP‐positive cells for PEI/pEGFP complex was ≈19% and was up to 28% for SS‐HPT/pEGFP complex, which were consistent with the results of the luciferase transfection assay (Figure 1d). We also examined the cellular uptake of PEI/pDNA and SS‐HPT/pDNA complexes in the H9c2 cells. The percentage of positive cells was ≈71% for PEI/pDNA and 93% for SS‐HPT/pDNA (Figure S5, Supporting Information). This increased cellular internalization ability of SS‐HPT, relative to PEI, had a positive influence on the transfection efficacy. We then used SS‐HPT and PEI coating *Drd5* siRNA or *Drd5* plasmid in H9c2 cells to measure the efficacy of delivery, via western blotting and RT‐PCR (Figure 1f,g). SS‐HPT provided a better delivery than PEI, as indicated by the greater decrease in D5R protein and mRNA expressions with SS‐HPT *Drd5* siRNA and the greater increase in D5R expression with SS‐HPT *Drd5* plasmid than their PEI counterparts. In vitro functional experiment showed that the silencing of D5R expression delivered by SS‐HPT in H9c2 cells led to hypertrophy, which was rescued by D5R over expression (Figure S6, Supporting Information). For the in vivo study, we constructed two *Drd5* plasmids with and without HA Tag. We found that D5R protein expression was not different between these two recombinant *Drd5* plasmids (Figure S7, Supporting Information). Therefore, the D5R overexpression in the heart (**Figure** **2**a–f) and liver (Figure S8, Supporting Information) is due to the recombinant protein delivered by SS‐HPT. In the in vivo study, we used SS‐HPT coated with *Drd5* plasmid without HA tag, because the addition HA tag may increase the instability of the complex and difficulty in entering the cell.^[^ ^30^, ^31^, ^32^ ^]^

### 2.2 SS‐HPT/*Drd5* Complex is Enriched in the Damaged Heart of Transverse Aortic Constriction Mice

It has been reported that the heart weight/body weight ratio and the biomarkers of cardiac hypertrophy (ANF, BNP) were significantly increased from post‐operative week 2 and remained significantly higher up to week 4 after thoracic aortic dissection or TAC, which induce left ventricular hypertrophy, but not heart failure.^[^ ^33^, ^34^ ^]^ Therefore the SS‐HPT/*Drd5* complex treatment was given for 4 weeks, 2 weeks after TAC surgery.

The SS‐HPT, conjugated with CY7, mixed with *Drd5* plasmid or *Drd5* siRNA, was injected into the tail vein in mice at 2 weeks after TAC or Sham control. The mice were sacrificed, and heart and kidney tissues were harvested at 6 weeks after TAC (Figure 2a). The cardiac enrichment of SS‐HPT/*Drd5* complex was examined by fluorescence emission tomography in SS‐HPT/*Drd5* complex injected mice. The fluorescence of the heart was about twofold higher in TAC mice compared with Sham control) mice, but fluorescence in the liver was similar in the two groups (Figure 2b–d). Positive nanostructured materials are endowed with the appropriate properties to go through physiological barriers, by effectively attaching to collateral vessels of the aorta, as is the case in the current study, allowing their transport into the cardiomyocytes.^[^ ^35^ ^]^ After TAC, rapidly proliferating endothelial cells have increased permeability which allows the leakage of the nanoparticles from the blood vessels, and consequently their entrance into the interstitial space and accumulation into the target organs, via endocytosis.^[^ ^36^, ^37^ ^]^ Western blotting and immunohistochemistry confirmed that D5R expression was downregulated in TAC group and further downregulated in the SS‐HPT/*Drd5* siRNA group but upregulated in SS‐HPT/*Drd5* plasmid group in the heart, yet such effects were not observed in the kidney (Figure 2e–h). We also found that D5R expression was downregulated in the SS‐HPT/*Drd5* siRNA group but upregulated in SS‐HPT/*Drd5* plasmid group in the liver (48 and 72 h post‐injection), but not in the spleen (at all three points) (Figure S8, Supporting Information). Fluorescence emission was very weak in the kidney and other organs in the TAC mice. Enhanced vascular permeability post operation promotes the tissue‐enrichment of SS‐HPT/NA complexes.^[^ ^25^ ^]^ These results confirmed that the SS‐HPT/*Drd5* complex was better enriched in the damaged heart than other organs and went through the liver for catabolism in the heart of TAC mice, which would be advantageous in the treatment of left ventricular hypertrophy.

### 2.3 Reversal of Left Ventricular Hypertrophy with SS‐HPT/*Drd5* Plasmid

**Figure 3:**
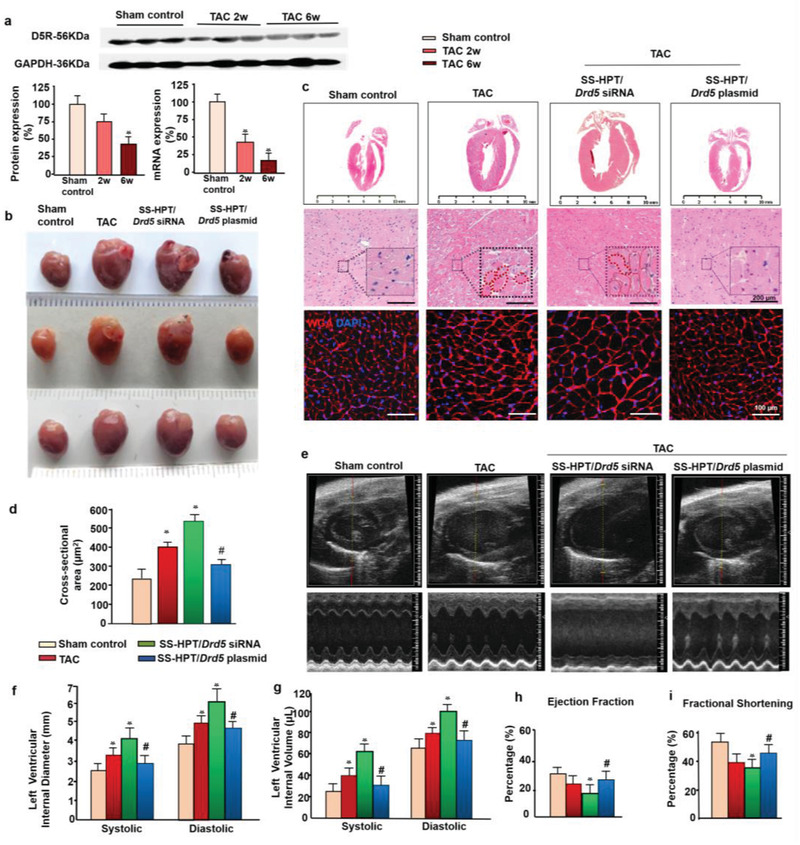
Reversible treatment of hypertrophic cardiomyopathy with SS‐HPT/*Drd5* complex. a) D5R protein and mRNA expression in hearts of mice 6 weeks post TAC quantified by immunoblotting, using GAPDH for normalization (*n* = 3 per group, *p < 0.05 vs Sham control). b) General phenotypic pattern captured by digital camera. c) Representative H&E staining of the whole‐heart transverse sections after thoracic aorta constriction (TAC) or SS‐HPT/*Drd5* complexes treatment (upper lane, scan bar is 10 mm). Magnification of H&E‐stained sections of the left ventricle (middle lane, scan bar is 20 µm). Wheat germ agglutinin‐Alexa Fluor 647 conjugates (red) staining of transverse sections of the left ventricular epicardial free walls, illustrating the membrane boundary of mouse myocytes. Original magnification, ×400 (lower lane, scale bar is 25 µm). d) Quantitative analysis of cross‐sectional area of the cardiomyocytes (*n* = 10 per group, *p < 0.01 vs Sham control, #*p* < 0.05 vs SS‐HPT/*Drd5* siRNA and TAC). e–i). Echocardiographic analysis of cardiac volume and function (*n* = 10 per group, *p < 0.01 vs Sham control, #*p* < 0.05 vs SS‐HPT/*Drd5* siRNA); one‐way ANOVA, Holm‐Sidak test. Data are presented as the mean ± SD from at least three independent experiments. TAC = transverse aortic constriction.

Mice with TAC had a time‐dependent decrease (0, 2, and 6 weeks) in cardiac D5R protein expression (**Figure** **3**a). We next checked the D5R expression in hearts of mice with left ventricular hypertrophy treated with SS‐HPT/*Drd5* plasmid or SS‐HPT/*Drd5* siRNA.

6 weeks after TAC, heart samples were obtained for analysis. As shown in Figure 3b–g, the hearts of TAC mice were bigger, with increased heart weight/body weight ratio (Figure S9a, Supporting Information), which were further increased in TAC mice treated with SS‐HPT/*Drd5* siRNA, relative to control mice (Figure 3b–g). By contrast, SS‐HPT/*Drd5* plasmid treatment mitigated the development of left ventricular hypertrophy and increase in heart weight/body weight ratio. Histopathology and WGA staining demonstrated that TAC mice had enlarged cardiomyocytes that were aggravated by SS‐HPT/*Drd5* siRNA and ameliorated by SS‐HPT/*Drd5* plasmid (Figure 3c,d).

Echocardiography showed significant hypertrophy and decreased ventricular diameter, 2 weeks after TAC, and hypertrophy and ventricular dilation, 6 weeks after TAC (Figure S10, Supporting Information). These effects were not only aggravated but also caused cardiac dysfunction by SS‐HPT/*Drd5* siRNA treatment. The SS‐HPT/*Drd5* siRNA mice, relative to TAC mice, had enlarged systolic and diastolic LV internal diameters and increased systolic and diastolic LV internal volumes (Figure 3e–g), as well as decreased LV ejection fraction and fractional shortening (Figure 3h,i). These effects were mitigated by SS‐HPT/*Drd5* plasmid. The body weight, water intakes, and energy intakes were similar in all groups (Tables S2 and S3, Supporting Information). The morphological and echocardiographic examinations indicated that the SS‐HPT/*Drd5* plasmid has a therapeutic effect in pressure overload‐induced left ventricular hypertrophy, but the mechanism warrants further exploration.

### 2.4 SS‐HPT/*Drd5* Plasmid Decreases Myocardial NADPH Oxidase Activity and Reactive Oxygen Species Generation

**Figure 4:**
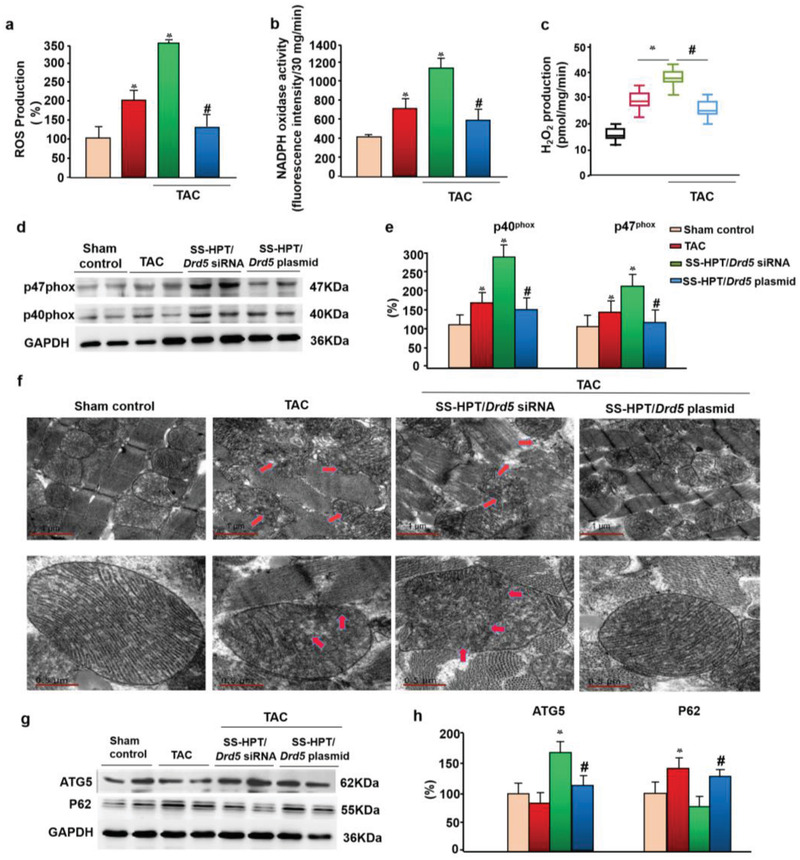
The regulation of myocardial reactive oxygen species production and autophagy by SS‐HPT/*Drd5* complex. a) ROS production was quantified by lucigenin chemiluminescence assay in the presence of NADPH (*n* = 10 per group, *p < 0.01 vs Sham control, #*p* < 0.05 vs SS‐HPT/*Drd5* siRNA and TAC). b) NADPH oxidase activity was quantified by lucigenin chemiluminescence assay. (*n* = 10 per group, *p < 0.01 vs Sham control, #*p* < 0.05 vs SS‐HPT/*Drd5* siRNA). c) H_2_O_2_ production from mitochondria isolated from mouse heart (*n* = 10 per group, *p < 0.01 vs TAC, #*p* < 0.05 vs SS‐HPT/*Drd5* siRNA). d,e) p40phox and p47phox protein expression quantified by western blot (*n* = 10 per group, *p < 0.01 vs Sham control, #*p* < 0.05 vs SS‐HPT/*Drd5* siRNA, one‐way ANOVA, Holm‐Sidak test). f) Myocardial mitochondrial morphology of mice observed by transmission electron microscope (TEMs, ×20 000); red arrows point to the TEMs. g,h) Autophagy markers expression (p62 and ATG5) in hearts of control, TAC, TAC‐SS‐HPT/*Drd5* plasmid treated mice; quantitative analysis using GAPDH for normalization, groups identified by color as in (a), (b), and (e). (*p < 0.05 vs Sham control, #*p* < 0.05 vs SS‐HPT/*Drd5* siRNA). Data are presented as mean ± SD.

Although the exact mechanisms that cause cardiac remodeling have not been fully elucidated, several have been proposed to contribute toward the development of myocardial remodeling, including oxidative stress^[^ ^38^, ^39^, ^40^, ^41^ ^]^ and autophagy.^[^ ^41^ ^]^ We have reported that the cardiac‐specific D5R deficiency in mice increased reactive oxygen species (ROS) production.^[^ ^14^ ^]^ Therefore, we determined whether the therapeutic effect of SS‐HPT/*Drd5* plasmid was caused by the decrease in oxidative stress.^[^ ^39^ ^]^ We found that ROS production and NADPH oxidase activity were increased by TAC and aggravated by SS‐HPT/*Drd5* siRNA but ameliorated by SS‐HPT/*Drd5* plasmid (**Figure** **4**a–c).

Some NADPH oxidase isoforms are the major sources of oxidative stress in many cells, including cardiac, vascular endothelial, and renal epithelial cells but not in cancer‐associated cachectic skeletal muscle cells.^[^ ^14^, ^39^, ^40^ ^]^ NADPH oxidases, depending on the particular isoform, are multi‐subunit enzymatic complexes consisting of membrane subunits gp91phox and p22phox and cytosolic subunits p47phox, p67phox, and p40phox.^[^ ^14^, ^39^, ^41^ ^]^ In our study, we found that membrane p40phox and p47phox proteins were increased in the hearts of TAC mice and further increased in SS‐HPT/*Drd5* siRNA mice. All of these effects of SS‐HPT/*Drd5* siRNA were completely blocked by the SS‐HPT/*Drd5* plasmid (Figure 4d,e). However, the percent increase in cardiac ROS production in TAC and SS‐HPT/*Drd5* siRNA mice was greater than the increase in NADPH oxidase activity. These could be taken to indicate that source(s) of ROS other than NADPH oxidases are involved in the increased ROS production in TAC mice.

In addition to the NADPH oxidases, mitochondria are important sources of ROS, including those in cardiomyocytes.^[^ ^42^, ^43^, ^44^ ^]^ We had found that D5R can decrease mitochondria‐induced ROS production in human renal proximal tubule cells (data not shown). To study further the effect of the D5R on the mitochondria‐induced ROS production in cardiomyocytes, we isolated the mitochondria and measured H_2_O_2_ production. Similar to the NADPH oxidase results, TAC increased mitochondria‐H_2_O_2_ production that was aggravated by SS‐HPT/*Drd5* siRNA; these effects were mitigated by SS‐HPT/*Drd5* plasmid (Figure 4c). It is believed that there is a “vicious cycle” between these two sources of ROS, that is, NADPH oxidase and mitochondria.^[^ ^45^, ^46^, ^47^ ^]^ To determine whether the mitochondria‐ROS is downstream to NADPH oxidase, we tested the mitochondrial function. In the TAC group, the mitochondria were swollen with irregular shapes, accompanied by vacuolization and mitochondrial crest rupture. These lesions were more extensive in the SS‐HPT/*Drd5* siRNA group. All of these abnormalities were reversed or improved by the SS‐HPT/*Drd5* plasmid, close to the control group (Figure 4f). We also found that JC‐1 red staining and Complex I and II‐III activities were decreased by TAC and further decreased by SS‐HPT/*Drd5* siRNA and almost normalized by SS‐HPT/*Drd5* plasmid (Figure S9b–d, Supporting Information). These data indicate that the SS‐HPT/*Drd5* siRNA can directly aggravate cardiac mitochondrial damage and increase mitochondria‐ROS production, and that SS‐HPT/*Drd5* plasmid therapy can effectively improve cardiac function by blocking mitochondria‐ROS production.

### 2.5 Autophagy Activation is a Double‐Edged Sword in SS‐HPT/*Drd5* Complex‐Treated Left Ventricular Hypertrophy

Autophagy, a conserved process that degrades dysfunctional proteins and cellular components, has been shown to play intricate and important roles in cardiac hypertrophy and heart failure,^[^ ^42^, ^45^, ^48^ ^]^ which can be both a cause and consequence of oxidative stress. ATG5 and P62 are used to monitor autophagy.^[^ ^49^, ^50^ ^]^ Dopamine increases autophagy in neuroblastoma cells. Activation of D5R, as with D3R and D4R, can increase autophagy protein expression and activation in neuroblastoma and human RPT cells^[^ ^51^, ^52^ ^]^ and murine kidney (unpublished data). 6 weeks of TAC slightly decreased ATG5 protein expression and increased P62 protein expression in the murine heart (Figure 4g,h). SS‐HPT/*Drd5* siRNA increased ATG5 expression above Sham and TAC mice and decreased the P62 expression. The increase in ATG5 expression caused by SS‐HPT/*Drd5* siRNA was decreased (normalized) by SS‐HPT/*Drd5* plasmid. The increase in P62 caused by TAC was decreased by SS‐HPT/*Drd5* siRNA but increased by SS‐HPT/*Drd5* plasmid close to the TAC level (Figure 3d–g). These results demonstrate that in the development of left ventricular hypertrophy, caused by TAC, D5R‐dependent increase in autophagy is a protective response in the murine heart. We have found that D5R‐regulated cardiomyocyte function is via protein kinase G (PKG) but not PKA in H9c2 cardiomyocytes (unpublished data). PKG1 can stimulate autophagy in cardiomyocytes by phosphorylation of TSC2 to suppress myocardial hypertrophy.^[^ ^53^, ^54^, ^55^ ^]^ In addition, PKG1 activation increases the expression of microtubule‐associated protein (LC3‐II) and the aggregation of myocardial markers, which is consistent with increased autophagy.^[^ ^56^ ^]^ During the development of left ventricular hypertrophy, D5R overexpression may activate PKG1‐induced autophagy, which can be an important protective mechanism in left ventricular hypertrophy.

Autophagy may be pivotal in maintaining normal myocardial morphology and cardiac function.^[^ ^47^ ^]^ However, the level of autophagy in the heart may determine whether it is beneficial or detrimental. Several studies have suggested that overactive and dysregulated autophagy may play a role in the transition from stable cardiac hypertrophy to decompensated heart failure.^[^ ^57^, ^58^ ^]^ In this study, TAC mice had deficiency of cardiac autophagy, evidenced by increased cardiac P62 and tendency for decreased ATG5, relative to controls. However, the SS‐HPT/*Drd5* siRNA‐treated TAC mice had overactive autophagy, with decreased cardiac P62 and increased cardiac ATG5, relative to the control (Figure 4g,h). These results indicate that overactive autophagy aggravates cardiac damage in SS‐HPT/*Drd5* siRNA mice, which have left ventricular hypertrophy and heart failure.

Therefore, in our study autophagy activation is a double‐edged sword, SS‐HPT/*Drd5* plasmid improvement of autophagy almost normalized cardiac function in TAC mice, but SS‐HPT/*Drd5* siRNA‐induced overactive autophagy may promote the transition from left ventricular hypertrophy to heart failure.

### 2.6 SS‐HPT/Drd5 Plasmid Prevents Cardiomyocyte Endoplasmic Reticulum Stress and Apoptosis

ER stress is an evolutionarily conserved cell response associated with numerous diseases, including cardiac hypertrophy and heart failure.^[^ ^59^ ^]^ During prolonged ER stress, increased apoptosis eliminates the unhealthy cells, which contributes to the process of cardiac hypertrophy,^[^ ^60^, ^61^ ^]^ may cause heart failure, arrhythmia, or sudden death.^[^ ^62^ ^]^ In this study, ER stress was also measured to analyze the effect of the SS‐HPT/*Drd5* plasmid. We found that markers of ER stress, ER oxidoreductin 1 (ERO1), binding immunoglobulin protein, hypoxia‐inducible factor 1, and C/EBP homologous protein (which can activate ERO1), were upregulated in the heart of TAC mice. The increased expressions of these proteins in TAC mice were further increased by SS‐HPT/*Drd5* siRNA but were decreased close to control values by SS‐HPT/*Drd5* plasmid treatment (Figure S11a–d, Supporting Information). The alleviation of the increase in ER stress markers by SS‐HPT/*Drd5* plasmid may indicate the involvement of ER in D5R‐mediated myocardial repair. Our study also suggests that D5R may improve myocardial function by decreasing cardiac ROS production, caused by NADPH oxidases and mitochondria, as well as ER stress.^[^ ^63^ ^]^

**Figure 5:**
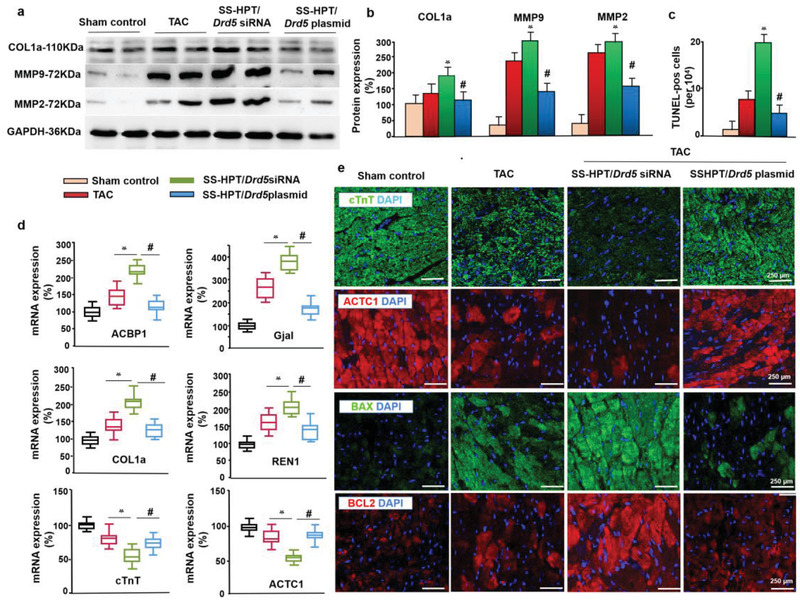
The regulation of cardiomyocyte apoptosis by SS‐HPT/*Drd5* complex. a,b) Western blot analysis of fibrosis markers, using cardiac protein extracts, including COL1a, MMP9, and MMP2 and quantitative analysis using GAPDH for normalization (*n* = 10 per group, *p < 0.05 vs Sham control, #*p* < 0.05 vs SS‐HPT/*Drd5* siRNA). c) Quantitative analysis of TUNEL‐positive myocardial cells (*n* = 10 per group, *p < 0.05 vs Sham control, #*p* < 0.05 vs SS‐HPT/*Drd5* siRNA). d) mRNA expression of myocardial markers quantified by PCR (*n* = 10 per group, *p < 0.05 vs Sham control, #*p* < 0.05 vs SS‐HPT/*Drd5* siRNA), one‐way ANOVA. e) Immunofluorescence analysis for cTnT(green), ACTC1 (red), and apoptosis markers including BAX (green) and BCL2 (red). Data are presented as the mean ± SD.

Accumulating evidence suggests that oxidative stress accelerates inflammation and apoptosis, which, in turn, causes cardiomyocyte hypertrophy and fibroblast proliferation, resulting in cardiac remodeling.^[^ ^64^ ^]^ We found that myocardial fibrosis markers (COL1a, MMP9, and MMP2) (**Figure** **5**a,b), hypertrophy markers (ANF and BNP) (Figure S11e,f, Supporting Information), DNA fragmentation (TUNEL test), and cardiac injury markers^[^ ^65^ ^]^ (ACBP1 and Gjal) were increased in the TAC‐mice and further increased in the SS‐HPT/*Drd5* siRNA mice, but were normalized or almost normalized in the SS‐HPT/*Drd5* plasmid mice (Figure 5a–d). Similar results were obtained for pro‐apoptotic protein, BAX, anti‐apoptotic protein, BCL2, and REN1 (Figure 5d,e); renin binds to cardiac receptors, insulin‐like growth factor II/mannose‐6‐phosphate, as part of the cardiac adaptation to stress caused by pressure overload.^[^ ^66^, ^67^ ^]^ In contrast to the increased expressions of the above proteins with TAC that were further increased by SS‐HPT/*Drd5* siRNA, the expressions of the cardiac regulatory protein, cardiac troponin T (cTnT, aka Tnnt2), and cardiac muscle *β*‐actin (AcTc1) were decreased by TAC, and aggravated by SS‐HPT/*Drd5* siRNA (Figure 5d,e). Cardiac troponins are used primarily for the diagnosis and management of acute myocardial infarction. cTnT is released into the circulation with a maximal elevation between 10 and 24 h post MI, and subsequently decreased at day 7.^[^ ^68^ ^]^ Therefore, we determined the myocardial cTnT and ACTC1 protein expression, and found that cTnT and ACTC1 protein expressions were elevated after 2 weeks of TAC but decreased close to normal after 6 weeks of TAC (Figure S12, Supporting Information). We also measured the serum cTnT and ACTC1 levels and found no significant differences in serum ACTC1 levels in the four groups. The cTnT levels increased after 4 weeks of TAC and were further increased by SS‐HPT/*Drd5* siRNA but was decreased close to sham by SS‐HPT/*Drd5* plasmid (Figure S12, Supporting Information). Therefore, serum cTnT levels may reflect the continuing myocardial injury better than protein expression. These findings provide evidence that SS‐HPT/*Drd5* plasmid can effectively repair cardiac damage and improve cardiac function. This mechanistic study demonstrates that SS‐HPT/*Drd5* plasmid ameliorates cardiac remodeling and function in mice with left ventricular hypertrophy. These findings could provide the rationale for D5R supplementary therapy for patients with left ventricular hypertrophy or heart failure.

### 2.7 Cardiac D5 Receptor and Autophagy Marker Expression in Patients with Hypertrophic Cardiomyopathy or Heart Failure Patient

HCM is a genetic disorder that is characterized by left ventricular hypertrophy, with nondilated ventricular chambers, unexplained by other causes. To explore the possibility for a clinical application of D5R supplementary therapy in HCM or left ventricular hypertrophy, we analyzed cardiac D5R and autophagy marker expression in 6 patients with presumed diagnosis of HCM, and 4 patients with heart failure. The baseline characteristics of both cohorts are shown in **Table** **1**.

**Table 1:** Baseline characteristics of the study population

	HCM (*n* = 6)	HF (*n* = 4)	*p‐*value
Demographics characteristics			
Age, y	34.6 ± 4.5	48.5 ± 5.1	0.121
Female sex, n (%)	2 (33.3%)	2 (50.0%)	0.008*
BMI, kg m^−2^	26.2 ± 3.1	27.5 ± 4.3	0.265
Clinical signs			
Systolic BP, mm Hg	132.2 ± 5.6	126 ± 8.1	0.113
Diastolic BP, mm Hg	82.6 ± 5.0	72 ± 14.1	0.378
Septal thickness (mm)	25.6 ± 14.0	10.5 ± 2.1	0.002*
Septal thickness range (mm)	16–50	8–12	
LV end diastole internal diameter (mm)	38.4 ± 2.7	71.5 ± 17.6	0.001*
LV end diastole internal diameter range (mm)	34–41	59–84	
LV thickness (mm)	14.6 ± 4.8	9.5 ± 2.1	0.023*
LV thickness range (mm)	11–23	8–11	
LVEF (%)	70.6 ± 5.1	25.5 ± 0.7	0.001*
LVEF range (%)	65–78	24–26	
Heart rate, bpm	81 ± 7.7	71.5 ± 8.8	0.167
Medical history, n (%)			
Hyperlipemia	0	2 (50%)	0.001*
Hypertension	3 (50%)	4 (100%)	0.008*
Medication, n (%)			
ACE‐inhibitor/ARB	0	4(100%)	0.001*
*β*‐Blocker	6 (100%)	4 (100%)	NA
Diuretic	0	4 (100%)	0.001*

Abbreviations: ACE = angiotensin‐converting enzyme; ARB = angiotensin II receptor blocker; BMI = body mass index; BP = blood pressure; bpm = beats/min; HF = heart failure; LVEF = left ventricular ejection fraction; NA, not available. *p‐value lower than the significance threshold of 0.05.

**Figure 6:**
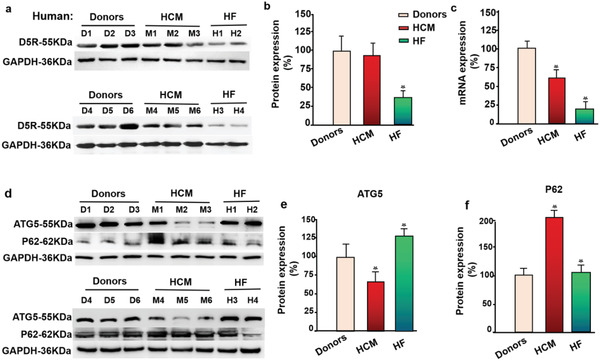
Cardiac D5R and autophagy markers in patients with HCM and heart failure. a) D5R and d) autophagy markers(P62, ATG5) expression in hearts of patients with hypertrophic cardiomyopathy(HCM) (*n* = 6), patients with heart failure (HF) (*n* = 4), and human donors (*n* = 6), quantified by b,e,f) immunoblotting, using GAPDH for normalization (*p < 0.05 vs donors). D5R mRNA expression c) in hearts of patients with HCM and heart failure quantified by RT‐PCR, using GAPDH for normalization (*p<0.05 vs donors), one‐way ANOVA, Holm‐Sidak test. Data are presented as mean ± SD.

The results showed that cardiac D5R protein expression was not decreased in patients with HCM but markedly decreased in the heart failure group, compared with the healthy subjects (donors) (**Figure** **6**a,b); (*n* = 6 in the HCM group; *n* = 4 in the heart failure group; *n* = 6 in the normal group (donors)). Cardiac D5R mRNA expression was decreased in the HCM group and decreased even more in the heart failure group (Figure 6c). Case‐review showed that the patients with HCM had taken the *β*‐blocker carvedilol orally for 3 to 5 months before Morrow septal myotomy. *β*‐blockers have been reported to inhibit the binding of Ang II to Ang II type 1 receptor (AT1R).^[^ ^69^ ^]^ However, carvedilol may inhibit the desensitization of the *β*‐adrenergic receptor (*β*AR) which is the crucial mechanism for cardiac damage.^[^ ^70^ ^]^ *β*‐arrestin is an important regulator in the desensitization of *β*ARs. Dopamine receptors are also linked to *β*‐arrestin. *β*‐arrestin/phospho‐ERK expression was significantly increased in D1R^−/−^ mice. However, the biased D1R agonist SKF83959 has a very low recruitment efficiency for *β*‐arrestin, in spite of increased cAMP production.^[^ ^71^ ^]^ It is possible that, *β*‐blockers may increase D5R expression by inhibiting AT1R and *β*‐arrestin signaling pathway. D5R and AT1R can decrease each other's expression.^[^ ^72^ ^]^

We then explored the mechanism of the cardiac dysfunction in these patients with HCM and heart failure. We found that in patients with HCM, cardiac ATG5 expression was decreased while cardiac P62 expression was increased (Figure 6d–f), similar to the results in the murine model of left ventricular hypertrophy (TAC‐induced) (Figure 4g,h). Patients with heart failure had increased ATG5 (Figure 6d), similar to TAC‐mice treated with SS‐HPT/*Drd5* siRNA. However, cardiac D5R protein expression in patients with HCM actually showed no significant difference from healthy donors, but was markedly decreased in heart failure patients (Figure 6a,b), similar to the end‐stage TAC mice and TAC mice treated with SS‐HPT/*Drd5* siRNA (Figure 2e–h).

We suggest that cardiac D5R may be critical for maintaining cardiac function and mitigating the development of left ventricular hypertrophy by improving autophagy. Our functional polycationic gene carrier system (SS‐HPT)‐coated *Drd5* plasmid is potentially useful in the treatment of left ventricular hypertrophy. We have reported that global *Drd5* knockout, in mice, leads to high blood pressure and cardiac hypertrophy.^[^ ^15^ ^]^ Aslam et al reported that 62% of their patients with HCM have hypertension.^[^ ^73^ ^]^ In our study, fifty percent of presumed HCM patients have hypertension (Table 1). Therefore, the efficacy of SS‐HPT/*Drd5* plasmid in the treatment of patients with HCM needs to be evaluated.

### 2.8 Biocompatible Properties of SS‐HPT/*Drd5* Complex

Organ toxicity of the different SS‐HPT/*Drd5* complexes was evaluated in vivo. No obvious pathological lesions were observed in the sections of the kidney, liver, lung, and spleen (Figure S13, Supporting Information). Any organ‐associated toxicity caused by the intravenous injection of different SS‐HPT/*Drd5* complexes in mice was also assessed by plasma biochemical parameters. As shown in Figure S14, Supporting Information, except for the test for cardiac toxicity (CK), the liver function tests (ALT, AST, and TBIL levels) and renal function tests (BUN and CRE levels) were similar in all the groups. CK was increased by TAC, and further increased by SS‐HPT/*Drd5* siRNA but normalized by SS‐HPT/*Drd5* plasmid. SS‐HPT contains tobramycin, an aminoglycoside antibiotic, used to treat infection. This component could enhance the biological safety for the delivery system. These data show that the SS‐HPT/*Drd5* complex is biocompatible in vivo, which further justifies its use in clinical studies in the future.

## 3 Conclusion and Perspectives

This work reports the beneficial effects of the SS‐HPT/*Drd5* plasmid complex in the therapy of pressure overload‐induced left ventricular hypertrophy and myocardial dysfunction by suppressing ROS production and regulating autophagy. We have also identified how the SS‐HPT/*Drd5* siRNA complex promotes the deterioration of left ventricular hypertrophy to heart failure by increasing oxidative stress and activating abnormal autophagy, leading to a vicious cycle in vivo.

Left ventricular hypertrophy is a well‐known independent risk factor for cardiovascular events. Persistent pathologic myocardial hypertrophy leads to myocardial remodeling and ultimately heart failure. Therefore, we explored the clinical significance of D5R in myocardial hypertrophy and heart failure based upon the animal study. We found cardiac D5R protein was slightly reduced in HCM patients and significantly decreased in heart failure patients. The patients with HCM (50% with hypertension) were on oral *β*1‐adrenergic receptor blocker (carvedilol) for 3 to 5 months before Morrow septal myotomy. The D5R, a member of the G protein‐coupled receptor family, negatively interacts with the AT1R.^[^ ^72^, ^74^ ^]^ D5R also negatively interacts with *β*‐arrestin, which may be upregulated by carvedilol treatment in HCM patients. Upregulated D5R may be one of the mechanisms to counteract the state of oxidative stress and improve autophagy but the eventual decrease in D5R expression may have facilitated the deterioration to heart failure.

Mechanistically, we found that TAC and TAC+ SS‐HPT/*Drd5* siRNA‐induced dysregulated autophagy and ROS generation may promote the transition from left ventricular hypertrophy to heart failure. Mitochondria‐ROS, via multiple signaling pathways, is involved in the regulation of autophagy.^[^ ^75^, ^76^ ^]^ The overall ROS production (but not NADPH oxidase activity or H_2_O_2_ production) was almost normalized by SS‐HPT/*Drd5* plasmid. We suggest that oxidative stress, especially in the mitochondria with disorganized autophagy, could have sped up the development of heart failure. The heart samples from patients of HCM and heart failure also showed dysregulated autophagy. The *β*‐adrenergic blocker carvedilol can trigger autophagy to inhibit the NLRP3 inflammasome.^[^ ^77^ ^]^ Although, the β‐adrenoceptor agonist, isoproterenol, has been reported to inhibit autophagy, *β*1‐adrenergic receptor autoantibodies have been shown to reduce autophagy.^[^ ^78^, ^79^ ^]^ Moreover, the combination of carvedilol and carnosic acid, an antioxidant, reduced ROS generation and autophagy in H9c2 cells with doxorubicin‐induced cardiotoxicity.^[^ ^80^, ^81^ ^]^ Masson et al had reported that left ventricular volume was decreased by the combination of an ACE inhibitor (delapril) and a D2R/*α*2 adrenergic agonist delivered through implanted osmotic mini‐pumps, but not by delapril alone in a model of chronic heart failure.^[^ ^82^ ^]^ Our multifunctional delivery of polycation hyperbranched polyaminoglycoside coated with *Drd5* plasmid (SS‐HPT/*Drd5* plasmid) normalized the dysregulated autophagy and reduced the ROS generation in TAC‐induced cardiomyopathy, which supports the importance and necessity of increasing D5R expression for left ventricular hypertrophy therapy.^[^ ^83^ ^]^

Collectively, our study demonstrates that reduced cardiac D5R expression is the key causative driver of the development of pressure overload‐induced left ventricular hypertrophy. We also show that supplementing D5R expression is important in maintaining normal heart function in mice with left ventricular hypertrophy and heart failure. The SS‐HPT/*Drd5* plasmid, with its biocompatibility and safety, can ameliorate cardiac remodeling and dysfunction caused by dysregulated autophagy and excessive ROS production. SS‐HPT/*Drd5* plasmid treatment in the early period of cardiac dysfunction may be a very promising therapeutic strategy to mitigate left ventricular hypertrophy and prevent or mitigate the development of heart failure. However, the efficacy of SS‐HPT/*Drd5* plasmid in the treatment of HCM needs to be determined; genetic studies are needed to examine the relationship between HCM and *DRD5* variants.

## 4 Experimental Section

Details on the synthesis and characterizations of SS‐HPT/NA (nucleic acids), procedures of cell viability assay, in vitro transfection assay, in vivo study in mouse myocardial hypertrophy models, and clinical cardiac sample assay, and any associated references are shown in the Supporting Information.

## Conflict of Interest

The authors declare no conflict of interest.

## Author Contributions

X.J., M.S., and X.L. contributed equally to this work. X.J.: Data analysis and writing of patient and animal study; M.S.: Data analysis and paper writing for functional polyaminoglycoside SS‐HPT study; X.L.: Performance of experiments and animal data analysis; X.L.: Animal surgery; X.Z.: Collection of patient samples; K.Y.: Patient clinical phenotype and genotype analysis; Y.W.: Patient sample data analysis; S.W.: Patient clinical data analysis; Y.Y.: Synthetic materials and quality testing; P.A.J.: Manuscript review; Z.Z.: Project design; F.‐J.X.: Manuscript review; Z.Y.: Project design and funding support.

## Supporting information

Supporting Information
