# Supplementary material for: Reversible Treatment of Pressure Overload‐Induced Left Ventricular Hypertrophy through Drd5 Nucleic Acid Delivery Mediated by Functional Polyaminoglycoside
Source: Adv Sci (Weinh). 2021 Jan 6;8(5):2003706. doi: 10.1002/advs.202003706 (PMC7927605; doi:10.1002/advs.202003706)
Supplement: Supplementary file 1 — Supporting Information [file ADVS-8-2003706-s001.pdf]

© 2020 Wiley-VCH GmbH

ADVANCED SCIENCE
Open Access

Supporting Information

for *Adv. Sci.,* DOI: 10.1002/advs.202003706

Reversible Treatment of Pressure Overload-induced Left Ventricular Hypertrophy Through Drd5 Nucleic Acid Delivery Mediated by Functional Polyaminoglycoside

Xiaoliang Jiang[1,†], Meiyu Shao[2,†], Xue Liu[1,†], Xing Liu[1], Xu Zhang[3], Yuming Wang[3], Kunlun Yin[4], Shuiyun Wang[5], Yang Hu[2], Pedro A Jose[6,7], Zhou Zhou[4], Fu-Jian Xu[2*], and Zhiwei Yang[1*]*

Supporting Information

Reversible treatment of pressure overload-induced left ventricular hypertrophy through *Drd5* nucleic acid delivery mediated by functional polyaminoglycoside

Xiaoliang Jiang[1,†], Meiyu Shao[2,†], Xue Liu[1,†], Xing Liu[1], Xu Zhang[3], Yuming Wang[3], Kunlun Yin[4], Shuiyun Wang[5], Yang Hu[2], Pedro A Jose[6,7], Zhou Zhou[4*], Fu-Jian Xu[2*], and Zhiwei Yang[1*]

[1]NHC Key Laboratory of Human Disease Comparative Medicine (The Institute of Laboratory Animal Sciences, CAMS&PUMC), and Beijing Engineering Research Center for Experimental Animal Models of Human Critical Diseases. 5 Pan Jia Yuan Nan Li, Chaoyang District, Beijing 100021, P. R. China.

[2]Key Lab of Biomedical Materials of Natural Macromolecules (Beijing University of Chemical Technology), Ministry of Education, Beijing Laboratory of Biomedical Materials, Beijing Advanced Innovation Center for Soft Matter Science and Engineering, Beijing University of Chemical Technology, Beijing 100029, P. R. China.

[3]Department of Hepato-Biliary-Pancreatic Surgery, Henan Provincial People's Hospital, People's Hospital of Zhengzhou University, Zhengzhou, Henan, 450003, P. R. China.

[4]Department of Cardiovascular Surgery, State Key Laboratory of Cardiovascular Disease, Fuwai Hospital, National Center for Cardiovascular Diseases, Chinese Academy of Medical Sciences and Peking Union Medical College, Beijing, 100037, P. R. China.

[5]Department of Pharmacology and Physiology, The George Washington University School of Medicine & Health Sciences, Washington, DC, 20052, USA.

[6]Department of Medicine, Division of Kidney Diseases & Hypertension, The George Washington University School of Medicine & Health Sciences, Washington, DC, 20052, USA.

[7]State Key Laboratory of Cardiovascular Disease, Beijing Key Laboratory for Molecular Diagnostics of Cardiovascular Diseases, Diagnostic Laboratory Service, Fuwai Hospital, National Center for Cardiovascular Diseases, Chinese Academy of Medical Sciences and Peking Union Medical College, Beijing 100037, P. R. China.

†These authors contributed equally to this work.

*Correspondence and requests for materials should be addressed to Z.W. Y. (email: yangzhiwei@cnilas.pumc.edu.cn) or to Z. Z. (email: zhouzhou@fuwaihospital.org) or to F.-J.X. (email: xufj@mail.buct.edu.cn).

Methods

Preparation and Physicochemical Characterization of SS-HPT/NA (nucleic acids):

The ring opening polymerization between hydroxyethyl disulfide diglycidyl ether and tobramycin resulted in the synthesis of hyperbranched SS-HPT (average molecular weight of 8.97×10^3 g mol^{-1}), as previously described [(S1)]. SS-HPT and NHS-CY7 (Solarbio, Beijing, P.R China) were incubated at room temperature (25°C) for 6 hours to synthesize SS-HPT-CY7, which was used in fluorescence emission-computed tomography (FLECT) imaging. The agarose gel electrophoresis assay, using a Sub-Cell system (Bio-Rad Laboratory, Hercules, CA, USA), was described in an earlier work [S1]. The particle sizes and zeta potentials of SS-HPT/NA nanocomplexes were measured in double distilled water or DMEM with or without 10% FBS (fetal bovine serum), via a Zetasizer Nano ZS (Malvern Instruments, Southborough, MA, USA). In addition, the morphological changes at the optimal weight ratio before or after $NaBH_4$ (100 mM) degradation were imaged by atomic

force microscopy (AFM, Bruker, USA). The detailed procedures were described in our previous work [S1].

Cell Culture and Biophysical Characterization of SS-HPT/NA:

Immortalized rat heart cells (H9c2) (Chinese Academy of Sciences for Type Culture Collection, Shanghai, China) were cultured in DMEM containing 10% FBS, 1% penicillin, and 1% streptomycin in an incubator with a temperature set at 37°C and 5% CO_2 atmosphere.

The cytotoxicity of SS-HPT was evaluated using a cell counting kit-8 (CCK-8, Dojindo Molecular Technologies, Shanghai, P. R. China). Transduction efficiency of SS-HPT in H9c2 cell lines was evaluated by using luciferase reporter gene (plasmid pRL-CMV encoding Renilla luciferase) and pEGFP-N1 reporter gene (encoding enhanced green fluorescent protein). The pDNA was labeled, in advance, by fluorescent dye YOYO-1. After incubation for 2 h, the polyethylenimine (PEI)/pDNA and SS-HPT/pDNA complexes at the optimal ratio were added into the 6-well plates, respectively. After 4 h of cellular uptake, the cells were washed three times with PBS to eliminate the extracellular fluorescence. Finally, the cells were imaged by a fluorescence microscope (Leica, DMI3000B, Germany) and counted by flow cytometry (MoFlo XDP, Beckman, USA). The H9c2 cells, 4×10^5, were seeded in a 6-well plates (NEST Biotechnology), and cultured for 24 h. When the cells were 90%-95% confluent, they were serum-starved for 2 h, and treated for 24 h in a

serum-free medium with phosphate-buffered saline, PEI/*Drd5* plasmid, PEI/*Drd5* siRNA, SS-HPT/*Drd5* plasmid, or SS-HPT/*Drd5* siRNA. The transfection efficiency of *Drd5* plasmid or *Drd5* siRNA mediated by SS-HPT or PEI was evaluated by reverse transcription quantitative polymerase chain reaction (RT-qPCR) assay and western blotting.

Murine model of TAC:

Adult (8-week-old) male C57Bl/6J mice were purchased from Beijing HFK Bioscience Co, Ltd. (Beijing, China). All animal-related studies were approved by the Institutional Animal Care and Use Committee of the Institute of Laboratory Animal Science [YZW2017005]. The animals were handled according to the guidelines and principles published in the National Institutes of Health Guide for the Care and Use of Laboratory Animals.

Cardiac pressure overload, to cause **left ventricular hypertrophy**, was induced by TAC in male mice aged 8 to 10 weeks [(S2)]. Mice anesthetized with 2% Avertin were placed in thermostatic heating pad (Hansen Medical Technology Company, Shandong, China), maintaining the animal body temperature between 36°C and 37°C throughout the procedure. A rodent ventilator (Harvard Apparatus, Holliston, MA, USA) was used to ventilate the animals which were intubated. To create pressure overload, the transverse aortic arch was constricted in the middle of the arch between the innominate and left common carotid arteries. The aortic arch was isolated by blunt

dissection, and a custom blunt needle (26.5 g) was positioned parallel to the aorta. A non-absorbable 7.0 nylon suture (Ethilon; Ethicon, Somerville, NJ, USA) was tied around the vessel and the needle, and then the needle was quickly withdrawn, and the incision closed, thereafter. Sham control (4-8 per group, unless otherwise indicated) mice were subjected to an identical procedure without the placement of a ligature. The intubation was removed after self-breathing was re-established. The animal was maintained on a heating pad until fully recovered. To measure cardiac D5R expression at different TAC periods, 3 samples were taken at each TAC stage from 3 different mice. The tissues were homogenized and frozen in −80°C (n = 9).

The mice that had TAC surgery (n = 12) were divided into three groups and treated with SS-HPT/*Drd5* siRNA (1.33 mg 2.5^{-1} $nmol^{-1}$), SS-HPT/*Drd5* plasmid (1.33 mg 2.5^{-1} $nmol^{-1}$), or vehicle (no treatment as the sham control) every 5 days from the second week to the sixth week by tail vein injection. At the end of the 4-week treatment, the mice were weighed, and echocardiographic images obtained. Then, the mice were anesthetized (2.5% Avertin, 0.012 mL g^{-1} body weight), and the hearts harvested, weighed, and studied.

FLECT/Computerized Tomography (CT):

FLECT imaging was performed using the Trifoil InSyTe FLECT® imager (beta version, TriFoil Imaging, USA). On the day of imaging, the nude mice (3 weeks after TAC surgery) were anesthetized, using isoflurane (4% for induction, 1.5% for

maintenance in 30% oxygen and 70% nitrous oxide), to obtain a three-dimensional image using 730-nm excitation laser and 803-nm filter. The absorption data were collected at 29 slices with 1-mm spacing and 29 source angles per slice. Reconstruction of the FLECT image was performed using 1 mm^3 grid with attenuation correction and 1,000 iterations to generate the reconstructed three-dimensional image of each scan. Following the FLECT scan, a CT scan was performed in each mouse using X-ray voltage of 45 kVp, exposure time of 1,500 ms, and over 360° projections to generate FLECT images, which were overlaid onto its respective FLECT image. FLECT and CT scan image files were then co-registered, fused, analyzed, and quantified using the analysis software In Vivo Scope version 2.00.

Transmission electron microscopy:

The myocardium was cut into a square (about 1 mm^3). After fixing with glutaraldehyde + osmium chloride and dehydrating, using gradient acetone solution, the square was embedded and cut into semi-thin sections. The sections were stained using a compound dye (0.25% sodium borate: 0.25% basic fuchsin = 1:1). After the tissues were microscopically examined, the sections were cut into ultrathin sections using a copper wire mesh attached to a film that was prepared with 0.45% Fonnvar solution. After staining at room temperature, using uranyl acetate and lead staining fluids, the samples were dried using a filter paper and then imaged in a JEM 1230

transmission electron microscope (JEOL USA Inc) at 110 kV and imaged with an UltraScan 4000 CCD camera and First Light Digital Camera Controller (Gatan Inc).

Echocardiography:

Echocardiography was performed as previously described (S3). The anesthetized (2.5% Isoflurane) mice were placed on a heating pads to keep the body temperature at 37 °C. The chests were shaved and covered with preheated echocardiography gel. Heart rate was monitored about 400-500 beats per minute and imaged with 30 MHz linear transducer (VisualSonics Vevo 770), with the mouse at a shallow left-side position. Two-dimensional left ventricular (LV) echocardiograms were obtained by placing the transducer along the long axis of the LV and directed to the right side of the neck of the mouse. Then, the transducers were rotated 90° clockwise and the LV short-axis views were visualized.

NADPH oxidase activity:

NADPH oxidase activity was assayed as previously described [S3]. A membrane and cytoplasmic protein extraction kit (Sangon Biotech Shanghai) was used to prepare the cardiac membranes. NADPH oxidase activity (light units per g protein per minute) of cardiac membranes was measured by NADPH-induced chemiluminescence with 5 μmol/L lucigenin and 100 μmol/L NADPH. Then, flavoprotein inhibitor diphenyleneiodonium (DPI) was used to verify the specificity of the NADPH-dependent O_2^- production.

Intracellular ROS and mitochondrial ROS measurements:

Intracellular and mitochondrial ROS were measured as previously described [(S3)]. For intracellular ROS, hydro-dichlorofluorescein diacetate (H_2DCF-DA) (Life Technologies) was used as probe. H9c2 cells or heart homogenates were collected after treatment and incubated with 100 mM Mito-tracker Red FM/ H2DCF-DA (dissolved in DMSO) for 30 min at 37 °C. The cells were then washed three times with PBS and the intracellular accumulation of fluorescent DCF-DA was measured by fluorescence microplate imaging (Carl Zeiss MicroImaging, NY). For mitochondrial ROS, isolated mitochondria were loaded with MitoSOX red (5 µM, for 10 min), according to the manufacturer's instruction. Sequential 2-D confocal images were taken at 405 nm excitation, and emission was collected at > 560 nm. To calculate the rate of MitoSOX signal change (dF/dt), fluorescent signals during a 5-min period before.

Mitochondrial isolation and measurement of mitochondrial potential:

Heart mitochondria were isolated from the rest of the homogenates, as previously described [S2, S3]. In brief, whole hearts, obtained from anesthetized mice, were immediately placed in ice cold isolation medium (250 mM sucrose, 10 mM Tris, 0.5mM EDTA PH 7.4 at 4 °C), then transferred to the tissue grinder for homogenization. An aliquot of the homogenates was snap frozen and stored at -80 °C rapidly. The mitochondrial membrane potential was measured by JC-1 staining

(T3168, formerly Life Technologies, Thermo Fisher Scientific, Waltham, MA, USA). After incubation with JC-1 dye, fluorescence at 485 nm was measured using a fluorometer (BioTek, Winooski, VT, USA), according to the manufacturer's instructions.

Histopathology:

After the mice were euthanized with an overdose of pentobarbital, tissues（heart, liver, lung, and spleen）were cut immediately. The heart, via its tip, was perfused with 4°C HTK solution (K^{+}=9 m $mol^{-1}L^{-1}$), at a constant perfusion pressure of 80 ± 2 mm Hg, using a peristaltic pump (Gilson Minipuls 4, Middleton, WI, USA). These tissues were perfused in 10% formalin, fixed, and stained, as previously described [S4]. Briefly, the fixed tissues were embedded in paraffin and cut serially from the apex to the base. The sections were stained with hematoxylin-eosin for histopathological analyses.

Immunoblotting, immunohistochemistry, and immunofluorescence:

Immunoblotting, immunohistochemistry, immunofluorescence staining, and electron microscopic examination were performed as previously described (S4). Briefly, the heart tissues obtained from the mice (Sham-Control, TAC, SS-HPT/*Drd5* siRNA, SS-HPT/*Drd5* plasmid) were lysed for immunoblotting or fixed in 10% formalin at a pH of 7.4. Dehydration, clarification, and inclusion were performed, soon afterward.

After blocks were obtained, 5 μm thick sections were obtained, using a microtome (Leica RM2235; Leica Microsystems). The protein samples were immunoblotted with well-characterized anti-p40phox (14648-1-AP, 1:500, Epitomics), anti-p47phox (28187-1-AP, 1:1000, Epitomics), anti-D5R (203-1-AP, 1:1000, Proteintech), anti-P62 (18420-1-AP, 1:1000, Proteintech), anti-ATG5 (10181-2-AP, 1:1000, Proteintech), anti-LC3II (12135-1-AP, 1:500, Proteintech), anti-MMP2 (10373-2-AP, 1:1000, Proteintech), anti-MMP9 (A2095, 1:500, Abclone), and anti-collagen 1 (COL1a) (ab34710, 1:500, Abcam) antibodies. Uniformity of protein loading, and membrane transfer were determined by immunoblotting for GAPDH. Primary antibody used in immunochemistry was anti-D5R (203-1-AP, 1:100, Proteintech). The primary antibodies used in immunofluorescence were anti-CTNT (15513-1-AP, 1:500, Proteintech), anti-ACTC1 (66125-1-lg, 1:100, Proteintech), anti-BAX (50599-2-lg, 1:500, Proteintech), and anti-BCL-2 (ab59348, 1:100, Abcam). Cardiac myocytes in paraffin-embedded heart sections were stained with wheat germ agglutinin (WGA) (red) Alexa Fluor^{TM} 647 Conjugate (w32466, Thermo Fisher). Rat cardiomyocytes (H9C2) were fixed, permeabilized, and stained with sarcomeric α-actinin (Sigma-Aldrich, #A7811) to measure cell size and DAPI to stain the nucleus. The size of cardiac myocytes was measured after WGA or α-actinin staining using a Zeiss measuring tool.

Quantitative real-time polymerase chain reaction (PCR):

Total RNA was purified using the RNeasy RNA Extraction Mini kit (Qiagen, Valencia, CA, USA). The RNA samples were converted into first strand cDNA using an RT2 First Strand kit, following the manufacturer's protocol (Qiagen). Quantitative gene expression was measured by real-time q-PCR, performed on an ABI Prism 7900 HT (Applied Biosystems, Foster City, CA, USA). The assay using gene specific primers (Qiagen) was performed, and SYBR Green real-time PCR detection method (Qiagen) was used as described in the manufacturer's manual. The primers used are shown in **Table S1**. The expressions of ACBP1, cTnT, COL1a, REN1, Actc1, Gial, ANF, and BNP were normalized to those of GAPDH and analyzed using the 2-ΔΔCt method.

Plasma biochemical measurements:

The plasma biochemical measurements were performed to identify hepatitis (determined by aspartate transaminase [AST], alanine transaminase [ALT], and total bilirubin [TBIL] levels), organ injury by blood urea nitrogen (BUN) and serum creatinine (CRE) levels (kidney), and creatine kinase (CK) level (heart) (AiDiKang Company, Beijing, P.R. China).

Human Cardiac Samples:

All heart samples obtained from humans followed the principles described in the

Declaration of Helsinki and approved by the ethics committee of Fuwai Hospital (2017-877). The left interventricular septum samples were obtained from six patients with obstructive HCM who had Morrow septal myectomy. Patients with congenital heart disease, myocardial infarction, or valvular heart disease were excluded from the study [S5, S6]. The control cardiac tissue samples were acquired from the same region of six healthy subjects who were brain-dead or died in accidents with hearts that were unsuitable for heart transplantation for noncardiac reasons [S7]. The heart samples of patients with heart failure came four heart transplant patients. Written informed consents were obtained from all of the participants or their relatives.

Statistical analysis:

The data are expressed as mean ± SD. Significant differences between and among the groups were determined using a one-way or a two-way factorial analysis of variance and Holm-Sidak test, for groups >2 and Student's *t*-test for groups = 2. The overall survival was analyzed using the Kaplan-Meier method and the log-rank test. A $p < 0.05$ was considered to be statistically significant. All of the statistical analyzes were performed using SPSS 22.0 statistical software (SPSS, Inc., Chicago, IL, USA).

S1a **S1b** **S1c** **S1d**

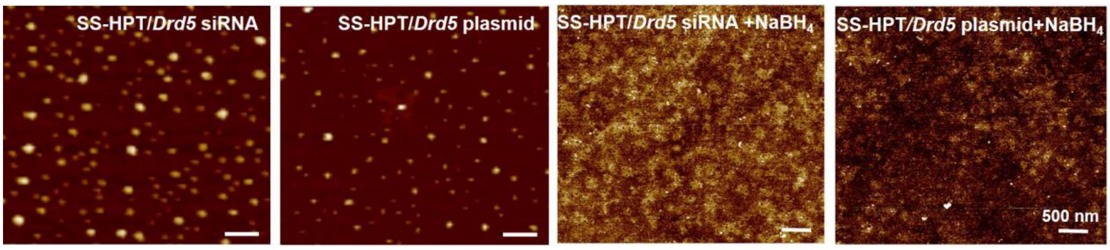

Figure. S1 Representative atomic force microscopy (AFM) images of SS-HPT/*Drd5* complexes. (**a-d**) Representative atomic force microscopy (AFM) images of SS-HPT/*Drd5* complexes at the typical weight ratio of 40 before or after degradation with $NaBH_4$.

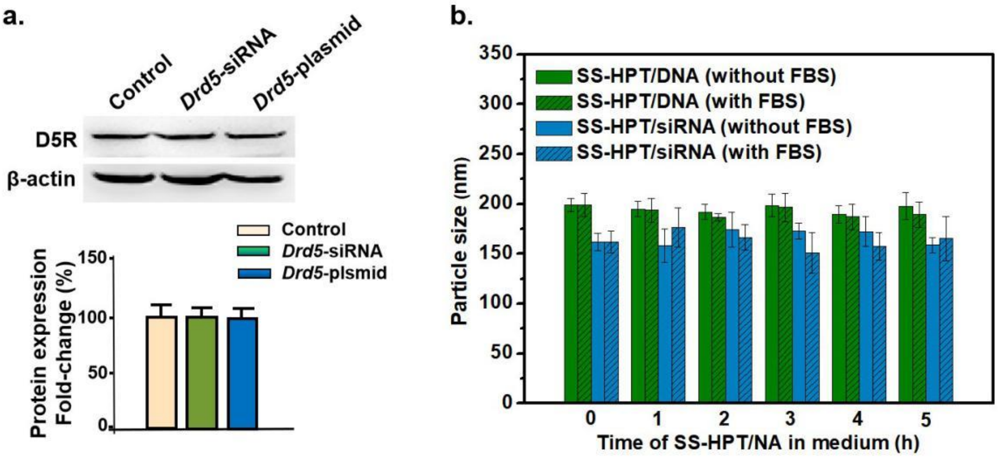

Figure. S2(a) D5R protein expression in the H9c2 cells transfected by *Drd5* siRNA and *Drd5* plasmid; quantitative analysis used β-actin for normalization (n=4/group, *P<0.05 vs control). **(b)** Particle sizes of SS-HPT/NA in cell culture medium. Particle sizes of SS-HPT/NA in cell culture medium (DMEM) without and with 10% serum (FBS) (*n*=6). The data are mean ± SD.

S3a **S3b**

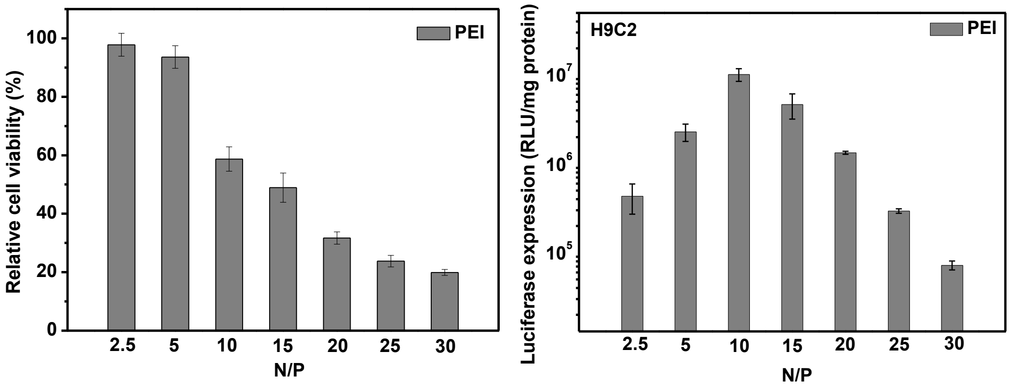

Figure. S3 Relative cell viability and luciferase expression with polyethylenimine. **a.** Relative cell viability and **b**. Luciferase expression with polyethylenimine (PEI, at various N/P ratios in H9c2 cells (25 kDa). (*n* = 6). The data are mean ± SD.

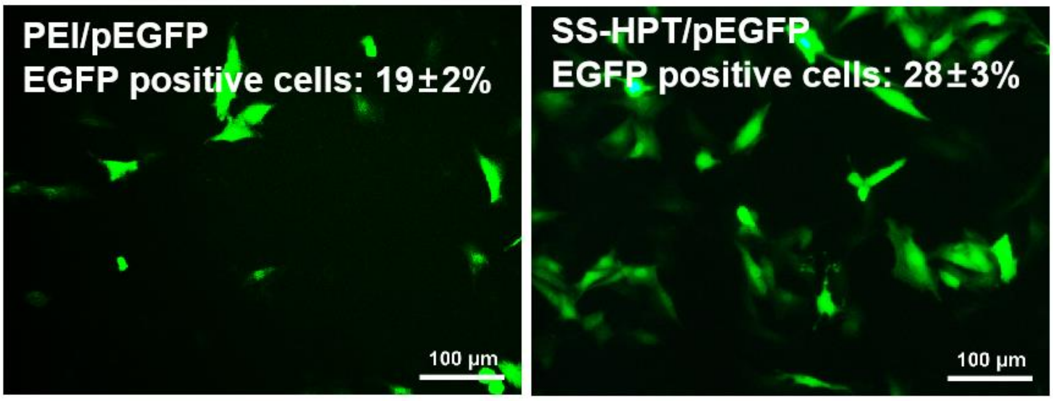

Figure. S4 Representative EGFP expression with PEI or SS-HPT treatment. Representative EGFP expression in H9c2 cells treated with SS-HPT at the optimal mass ratio of 40 compared with PEI at the optimal N/P ratio of 10. The data are mean ± SD.

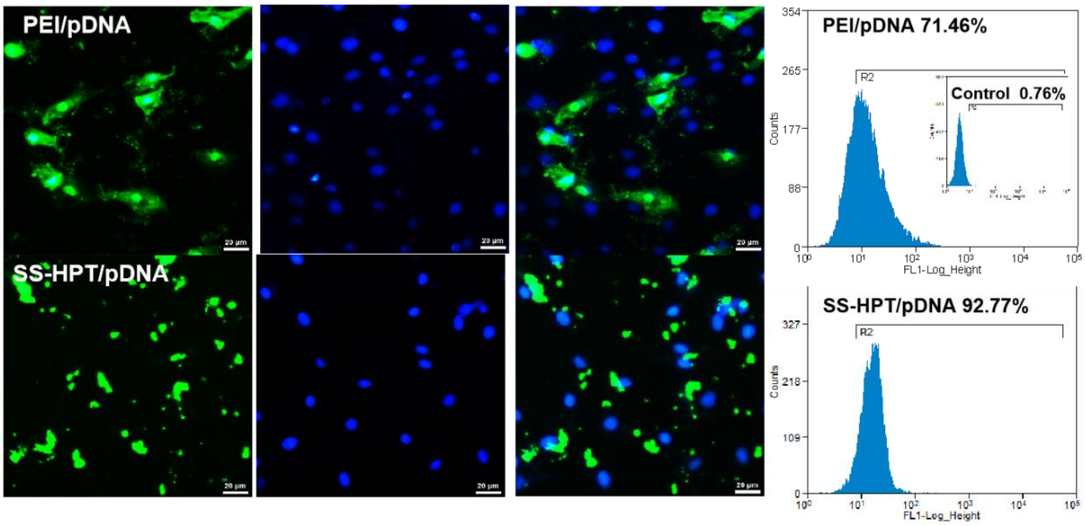

Figure. S5 Intracellular endocytosis ratio of PEI/pDNA and SS-HPT/pDNA. Cellular internalization of H9c2 cells treated with PEI and SS-HPT/pDNA at the optimal ratio; YOYO-1-labeled pDNA is shown in green and the DAPI-labeled nucleus is shown in blue.

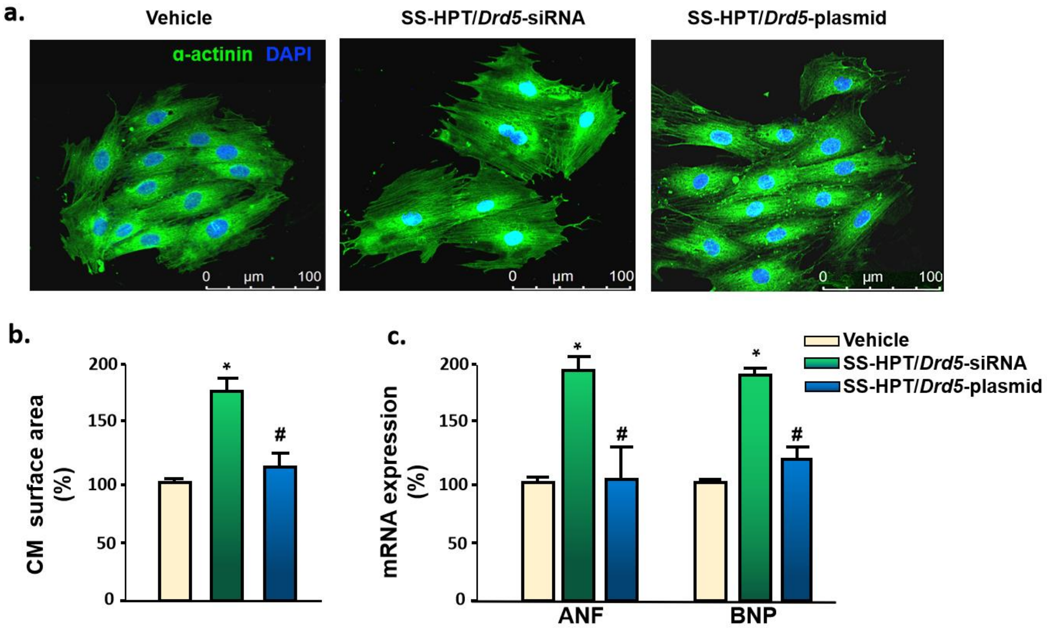

Figure. S6 (a, b) Immunofluorescent staining for α-actinin (green) to quantify the cardiomyocyte size; nuclei were counter stained with DAPI (blue). Original magnification ×400, scale bar is 100 μm (n=4). **(c)** mRNA expression of ANF and BNP quantified by RT-qPCR in the different groups (n=6/group, *P < 0.05 vs vehicle, #P < 0.05 vs SS-HPT/*Drd5* siRNA), one-way ANOVA, Holm-Sidak test. The data are mean ± SD.

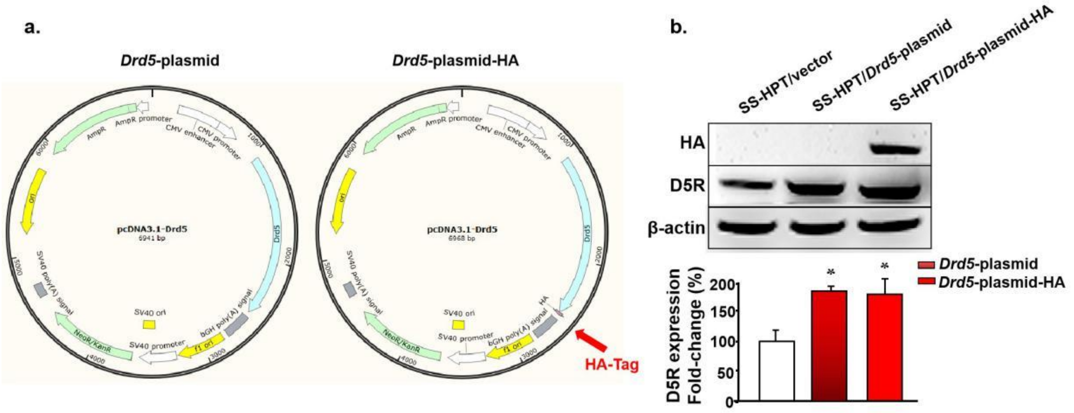

Figure. S7 (a) Sketch map of the *Drd5* plasmid construct with or without HA tag. **(b)** HA and D5R expression in *Drd5* plasmid transfected H9c2 cells; quantitative analysis used β-actin for normalization (n=4/group). *P < 0.05 vs SS-HPT/vector, one-way ANOVA, Holm-Sidak test. The data are mean ± SD.

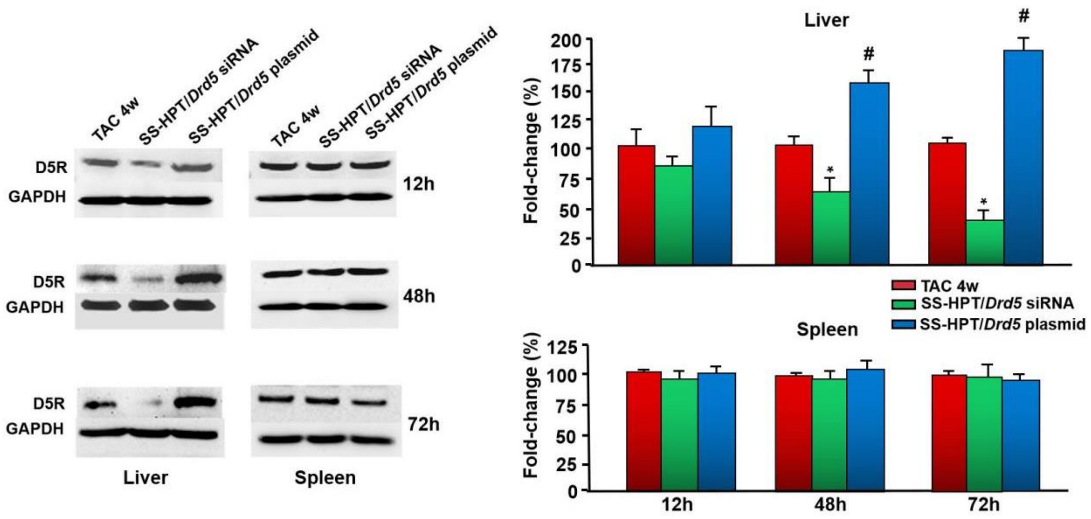

Figure S8. *In vivo* distribution of SS-HPT/*Drd5* complex in liver and spleen. D5R protein expression in the liver and spleen from TAC mice 4 weeks post SS-HPT/*Drd5* complex injection at three points (12h, 48h, and 72h); quantitative analysis used GAPDH for normalization (n = 4/group, *P < 0.01 vs TAC mice, $^{\#}$P < 0.05 vs others, one-way ANOVA, Holm-Sidak test. The data are mean ± SD.

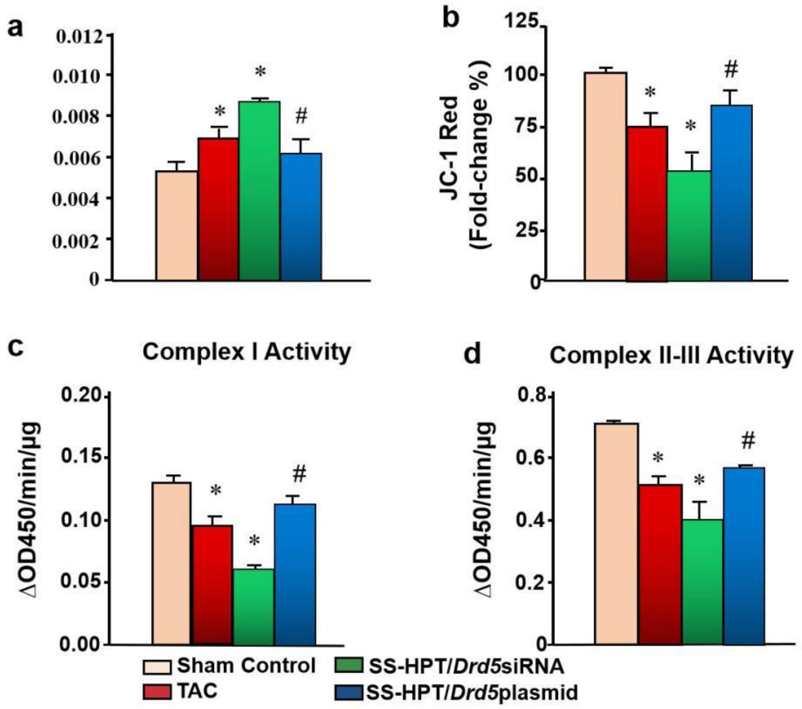

Figure. S9 SS-HPT/*Drd5* plasmid improves mitochondrial activity evaluated by JC-1 Red staining and measurement of Complex 1 and Complex II-III activities. **(a)**. Heart weight and body weight ratio in the four groups of mice. **(b)**. Fold-changes of JC-1 Red in the four groups of mice. **(c-d)** Fold-changes of Complex I and Complex II-III activities in the four groups of mice. (n=10/group, *P<0.05 vs Sham control, $^{\#}$P<0.05 vs SS-HPT/*Drd5* siRNA), one-way ANOVA, Holm-Sidak test. Data are presented as mean ± SD.

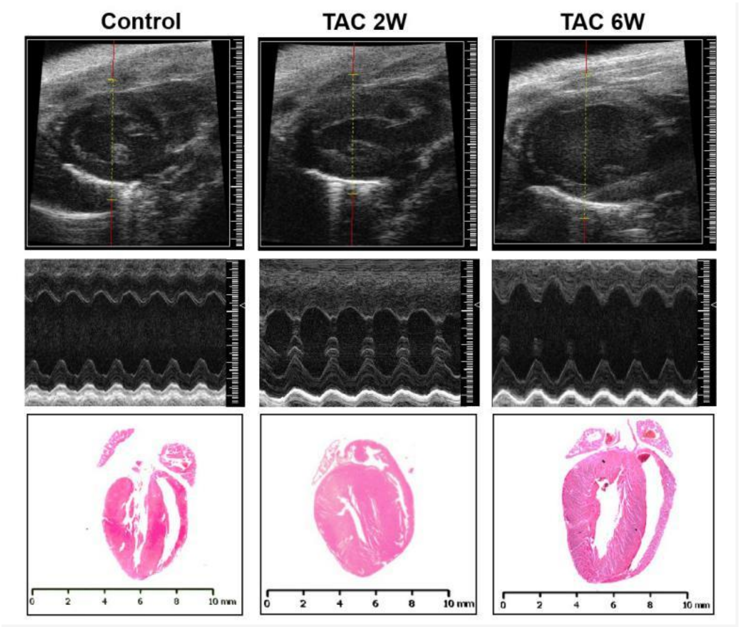

Figure S10. Echocardiographic analysis (upper and middle lanes) and representative H&E staining (lowest lane) of cardiac morphology in control and TAC groups (n=10/group). Images are from one of three independent experiments.

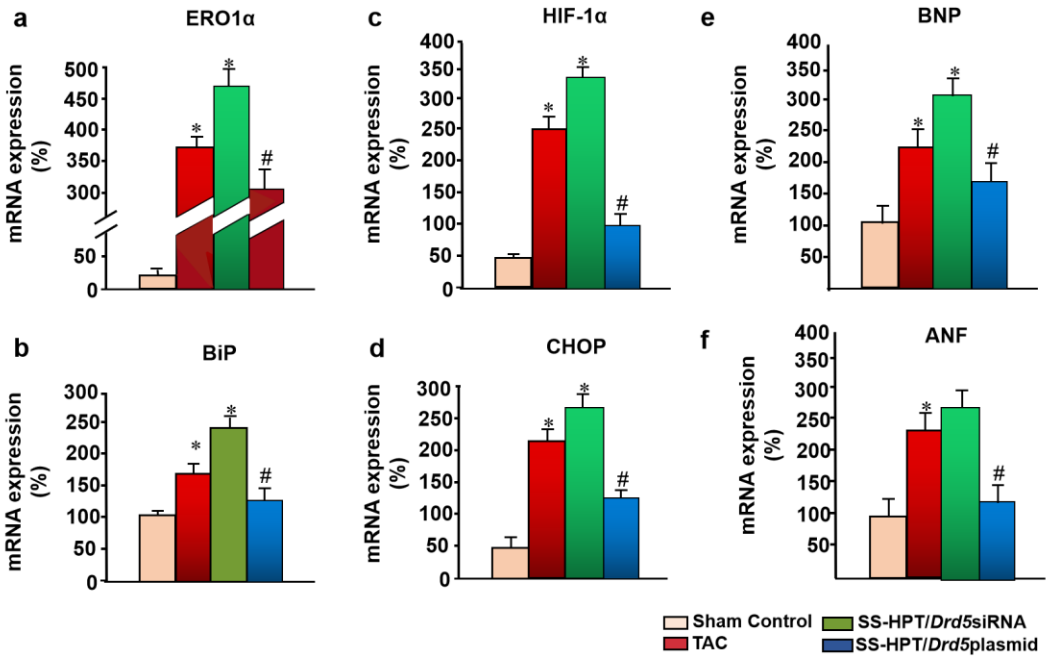

Figure. S11 SS-HPT/*Drd5* plasmid prevents cardiomyocyte ER stress. The mRNA expressions of endoplasmic reticulum stress markers (ERO1, HIF1α, BiP, and CHOP) **(a-d)** and hypertrophy markers (ANF and BNP) **(e-f)** in mouse heart were quantified by RT-qPCR, and corrected by GAPDH mRNA expression (*n*=10/group, *P<0.05 vs Sham control, #P<0.05 vs SS-HPT/*Drd5* siRNA), one-way ANOVA, Holm-Sidak test. Data are presented as mean ± SD.

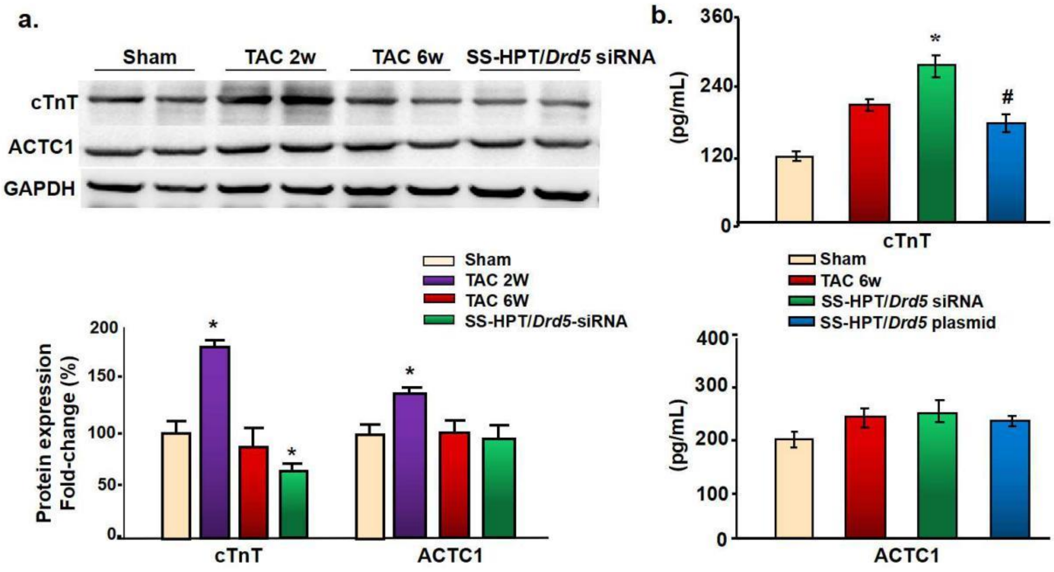

Figure. S12 (a) Western blot analysis for cTnT and ACTC1, and quantitative analysis using GAPDH for normalization (n=6/group, *P < 0.05 vs Sham). **(b)** Plasma biochemical measurements of cardiac troponin T (cTnT) and cardiac muscle β-actin (ACTC1) (#P < 0.05 vs SS-HPT/*Drd5* siRNA group). one-way ANOVA, Holm-Sidak test. Data are presented as mean ± SD.

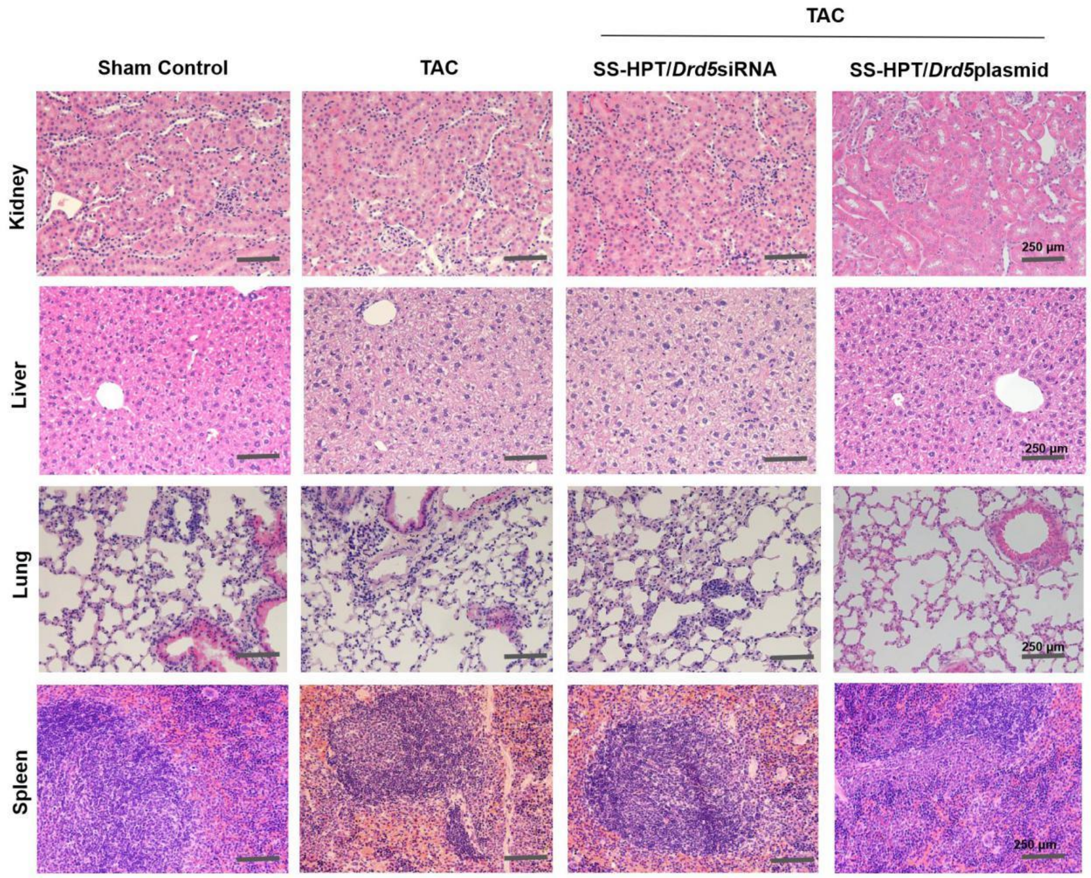

Figure. S13 Representative photographs of H&E staining of paraffin-embedded sections of the four organs.

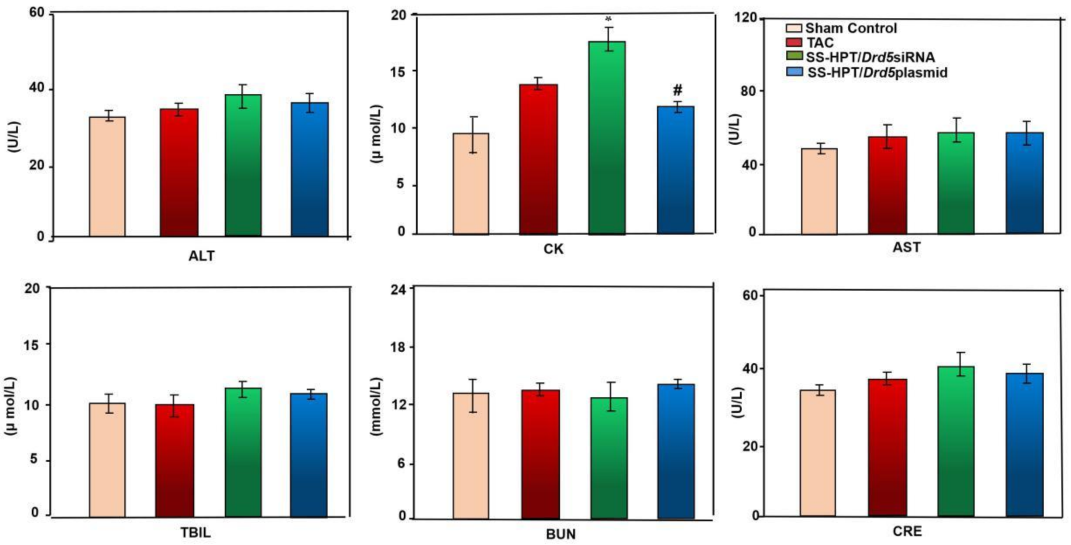

Figure. S14 Plasma biochemical measurements of alanine transaminase (ALT), creatine kinase (CK), aspartate transaminase (AST), total bilirubin (TBIL), blood urea nitrogen (BUN) and serum creatinine (CRE). (*P < 0.05 vs Sham, #P < 0.05 vs SS-HPT/*Drd5* siRNA group, n =10). one-way ANOVA, Holm-Sidak test. Data are presented as mean ± SD.

	**Primer (F)**	**Primer (R)**
ACBP1	GTGTTTATGGATTTGCTTCCTCGG	TGCAGTAGTCCCAGAGGTCA
cTnT	CGCTGACTTCTGTCACACAC	GAGGTCAGGAGGCTTGGCATCT
COL1a	CATTTCCGCCTCTGGCGAATG	CACATAAGGCCCAGAAGCCT
REN1	TTTCCGCCTCTGGCGAATG	AGATCGTCCATGGTCAGGGT
Actc1	GAACCTCAGTCACCGTCTCC	CATCACAACATCCGATCCGC
Gial	ATTTCCGCCTCTGGCGAAT	TTGCTTCGGCTGTTCGATGA
ANF	TGATGGATTTCAAGAACCTGCT	GATCTATCGGAGGGGTCCCA
BNP	CAGAAGCTGCTGGAGCTGATA	TCCGGTCTATCTTCTGCCCA

Table S1 Primer information for Drd5, inflammation factors, and myocardial markers.

	**Sham Control**	**Mock**	***Drd5* siRNA**	***Drd5* plasmid**
**1st week**	16.61±2.06	17.23±1.39	18.71±3.21	17.56±3.55
**2nd week**	17.40±4.17	18.80±1.91	19.43±2.65	18.83±2.45
**3rd week**	20.32±3.30	20.60±4.52	20.38±3.13	20.43±3.34

**4th week**	22.60±5.73	21.10±7.82	22.05±2.69	21.91±2.13
**5th week**	24.57±4.19	23.75±7.83	24.57±2.47	25.33±2.09
**6th week**	25.33±4.30	25.60±6.40	26.71±2.38	26.57±2.55

Table S2 Body weights of Sham control mice and TAC-mice treated with mock, SS-HPT/*Drd5* siRNA, or SS-HPT/*Drd5* plasmid.

	**0w**	**TAC 2w**	**TAC 4w**	**TAC 6w**
**Water intake (mL day^{-1})**				
**Sham Control**	5.82±0.12	5.85±0.10	5.88±0.23	5.87±0.20
**Mock**	5.83±0.27	5.86±0.08	5.86±0.25	5.88±0.16
***Drd5* siRNA**	5.72±0.14	5.67±0.11	5.87±0.28	5.07±0.10
***Drd5* plasmid**	5.43±0.21	5.43±0.09	5.39±0.20	5.28±0.36
**Energy intake (g day^{-1})**				
**Control**	4.63±0.23	4.65±0.19	4.68±0.27	4.70±0.31
**Mock**	4.48±0.23	4.52±0.09	4.53±0.26	4.63±0.17
***Drd5* siRNA**	4.34±0.20	4.03±0.10	4.44±0.32	4.90±0.01
***Drd5* plasmid**	4.51±0.22	4.57±0.34	4.53±0.06	4.23±0.37

Table S3 Water and energy intakes of Sham control mice and TAC-mice treated with

mock, SS-HPT/*Drd5* siRNA, or SS-HPT/*Drd5* plasmid.

References

[S1] Y. Huang, X. Ding, Y. Qi, B. Yu, F. J. Xu, *Biomaterials.* **2016**, 106, 134.

[S2] G. Karamanlidis, C. F. Lee, L. Garcia-Menendez, S. C. Kolwicz, Jr., W. Suthammarak, G. Gong, M. M. Sedensky, P. G. Morgan, W. Wang, R. Tian, *Cell Metab.* **2013**, 18, 239.

[S3] X. Jiang, Y. Liu, X. Liu, W. Wang, Z. Wang, Y. Hu, Y. Zhang, Y. Zhang, P. A. Jose, Q. Wei, Z. Yang, *Redox Biol.* **2018**, 19, 134.

[S4] X. Ma, K. Takeda, A. Singh, Z. X. Yu, P. Zerfas, A. Blount, C. Liu, J. A. Towbin, M. D. Schneider, R. S. Adelstein, Q. Wei, *Circ Res.* **2009,** 105, 1102.

[S5] K. Chen, L. Gao, Y. Liu, Y. Zhang, D. S. Jiang, X. Wei, X. H. Zhu, R. Zhang, Y. Chen, Q. Yang, N. Kioka, X. D. Zhang, H. Li, *Basic Res Cardiol.* **2013**, 108, 338.

[S6] X. Zhu, J. Fang, D. S. Jiang, P. Zhang, G. N. Zhao, X. Zhu, L. Yang, X. Wei, H. Li, *Hypertension.* **2015**, 66, 571.

[S7] Y. Zhang, Y. Liu, X. H. Zhu, X. D. Zhang, D. S. Jiang, Z. Y. Bian, X. F. Zhang, K. Chen, X. Wei, L. Gao, L. H. Zhu, Q. Yang, G. C. Fan, W. B. Lau, X. Ma, H. Li, *Cardiovasc Res.* **2014**, 102, 35.
